# Two new species of *Princaxelia* (Crustacea, Amphipoda, Pardaliscidae) from hadal depths of the Tonga and Mariana trenches (Pacific Ocean)

**DOI:** 10.3897/zookeys.1289.204655

**Published:** 2026-08-14

**Authors:** Grady A. Duffy, Jennifer A. Wainwright, Brett C. Gonzalez, Todd Bond, Alan J. Jamieson

**Affiliations:** 1 Minderoo-UWA Deep-Sea Research Centre, School of Biological Sciences and Oceans Institute, The University of Western Australia, 35 Stirling Highway, Perth, WA, 6009, Australia Minderoo-UWA Deep-Sea Research Centre, School of Biological Sciences and Oceans Institute, The University of Western Australia Perth Australia

**Keywords:** Crustacean, deep-sea, deep-sea ecology, integrative taxonomy, invertebrate zoology

## Abstract

*Princaxelia* Dahl, 1959, is a genus containing predatory pardaliscid amphipods, typically captured in small numbers from hadal trenches (> 6,000 m ocean depth). Here, two new species, *Princaxelia malohi* Duffy & Wainwright, **sp. nov**. and *Princaxelia kahat* Duffy & Wainwright, **sp. nov**., are added to the genus based on key morphological features to bring the total number of species of *Princaxelia* to seven. DNA barcodes at the 16S and COI region are also provided for all type specimens where possible to add to the limited but growing number available. *Princaxelia malohi***sp. nov**. was described from five specimens captured using baited landers between 8,200–8,350 m water depth in the Tonga Trench. They exhibit the first identified record of *Princaxelia* from this hadal feature. Key diagnostic features include the presence of two plumose setae on the inner plate of maxilla 1 and one projection near the base of the dactylus in gnathopods 1 and 2. The second species, *Princaxelia kahat***sp. nov**., is described from between 8,000–8,964 m in the Mariana Trench, and is the second species described from this hadal feature. Key diagnostic features for this species include the presence of six plumose setae on the inner plate of maxilla 1, and three to four projections near the base of the dactylus in gnathopod 1, and four to five projections on the base of the dactylus in gnathopod 2. A morphological matrix and key to the genus is updated to aid in future identification of species of *Princaxelia* and notes on *in situ* observations are also included.

## Introduction

The hadal zone is the deepest of the oceanic biozones, comprising areas that lie from 6,000 to almost 11,000 m deep, and make up 45% of the oceans total depth range. Areas that reach these extreme depths are shaped by geological activity and tectonic plate movement, forming unique features such as trenches and fracture zones that are often isolated from each other (Jamieson and Stewart 2021). This isolation, especially when coupled with extreme hydrostatic pressures, makes the hadal zone an intriguing ecosystem for understanding the diversity of organisms and genetic connectivity between ultra deep systems (Jamieson et al. 2010).

Scavenging amphipods are one of the most prominent groups of organisms at hadal depths (Dahl 1959; Wolff 1959; Hessler et al. 1978). These scavengers are in such abundance that they are a key prey resource for larger organisms (Jamieson and Weston 2023; Wainwright et al. 2026). For example, above ~8,200 meters, large teleosts (bony fish) such as snailfish (Liparidae) and cusk-eels (Ophidiidae) are known to prey upon scavenging amphipods (Jamieson et al. 2010; Dasgupta et al. 2024) whereas at deeper depths, the top predators shift to invertebrate groups. Predatory hadal amphipods have known representatives from the families Eusiridae (Lörz et al. 2018; Weston et al. 2024) and Pardaliscidae (Jamieson et al. 2012) and are less frequently caught in baited traps tailored for scavenging species due to their preference for live prey. Baited traps are currently the most common biological hadal sampling method (Jamieson et al. 2013), and as such the resulting lack of captures of predatory amphipods provide a gap in understanding of the true diversity of non-scavenging amphipods (Weston et al. 2024). *In situ* observations of amphipod predation events by other amphipods have primarily been observed from the genus *Princaxelia* (Jamieson et al. 2012).

*Princaxelia* Dahl, 1959 is a pardaliscid amphipod primarily known from hadal trenches across the western Pacific (Kamenskaya 1981). They are distinguished from other pardaliscid amphipods by a large, elongated maxilliped palp that extends over twice the length of the outer plate (Karaman 1974). There are currently five species described within the genus: *Princaxelia abyssalis* Dahl, 1959 initially described from the Kermadec Trench, with further reports from the Aleutian, Kuril-Kamchatka, Izu Bonin (Izu-Ogasawara), Yap, Japan, Philippine, and Bougenville trenches at depths between 6,435 and 9,530 m (Kamenskaya 1981); *Princaxelia magna* Kamenskaya, 1977 from the Yap Trench at depths between 7,190 and 7,250 m; *Princaxelia jamiesoni* Lörz, 2010 initially described from the Japan and Izu-Ogasawara trenches, but further reported from the Kuril-Kamchatka Trench at depths between 7,703 and 9,316 m (Jażdżewska and Mamos 2019); *Princaxelia marianaensis* Tomikawa & Watanabe in Tomikawa, Watanabe, Tanaka & Ohara, 2021 from the Mariana Trench at 5,683 m depth; and *Princaxelia stephenseni* Dahl, 1959, the type specimen, from the northwest Atlantic off the coast of Iceland at a depth of 1,505 m (Stephensen 1931). Being a predatory genus, the lack of captured specimens has caused difficulties and uncertainties in the taxonomy of *Princaxelia*.

This study describes two new species; the first species, *Princaxelia malohi* sp. nov., is described using five specimens captured between 8,000–8,350 m in the Tonga Trench (SW Pacific Ocean). Supplemental *in situ* footage from *Princaxelia* species from this expedition is also provided. The second species, *Princaxelia kahat* sp. nov., is described from the Mariana Trench (NW Pacific Ocean) at depths between 8,000–8,964 m. These represent the sixth and seventh species described within the genus, with *Princaxelia malohi* sp. nov., constituting the first identified occurrence of *Princaxelia* from the Tonga Trench.

## Materials and methods

### Study site and specimen collection

**Figure 1.**
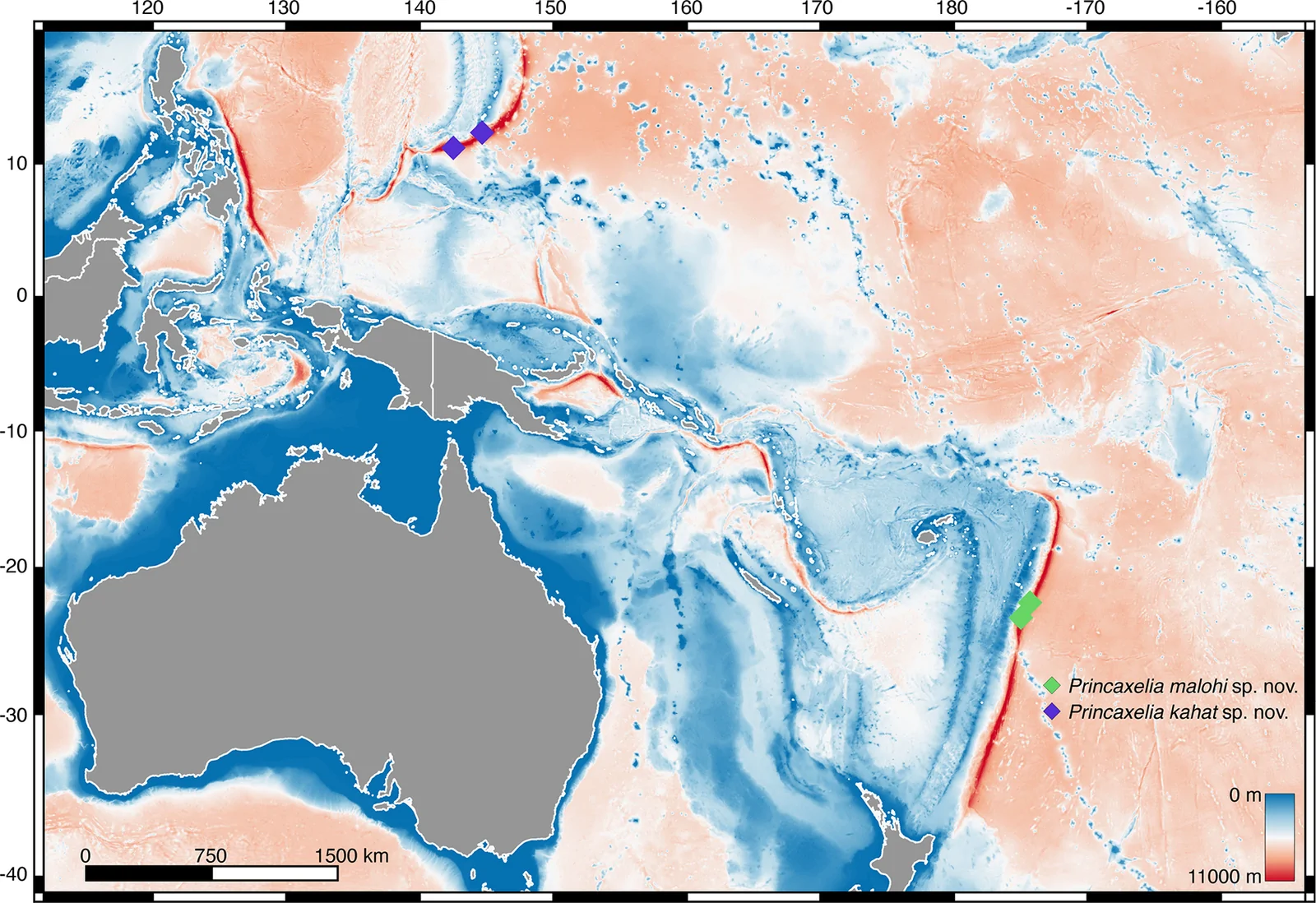
Map of study sites. *Princaxelia malohi* sp. nov. (green diamond) collected from the Tonga Trench during the 2024 Inkfish Tonga Trench Expedition aboard RV Dagon; *Princaxelia kahat* sp. nov. (blue diamond) collected from the Mariana Trench deployed from the vessels RV Falkor (FK 141109) in 2014 and TV Shinyo-Maru (SY1615) in 2017.

All amphipod specimens from were collected using funnel traps baited with mackerel (Scombridae) and attached to free fall landers, as described in Jamieson et al. (2013). Landers were deployed during the 2024 Inkfish Tonga Trench Expedition aboard RV Dagon (Fig. 1). Specimens were fixed immediately in 99–100% ethanol. Ethanol was replaced after 24 hrs and samples were stored at −12 °C. Collected specimens were sorted and logged on Dagon and further sorted and stored at room temperature in the Minderoo-UWA Deep-Sea Research Centre collection until taxonomic examination.

Specimens from the Mariana Trench were collected from landers deployed from the vessels RV Falkor (FK 141109) in 2014 (Drazen 2015) and TV Shinyo-Maru (SY1615) in 2017 (Fig. 1). Specimens were fixed in 99–100% ethanol and stored in the Minderoo-UWA Deep-Sea Research Centre collection.

### Morphological examination

Specimens were imaged using a Canon EOS R5 DSLR camera with a Canon 100 mm f/2.8 VC USD Macro 1:1 VC lens. Images were stacked using the software Helicon Focus v.8.3.7 (Helicon Soft). Specimens were dissected in 99% ethanol under a Leica M205C dissecting microscope. The limbs on the left side of the specimens were used for dissection to keep consistent with prior descriptions. Select body parts were stained using Shirlastain-A fibre identification stain to help identify finer morphological features. Dissected appendages were mounted onto microscope slides using glycerol and imaged using a Nikon Eclipse Si compound microscope and a mounted Tuscen True chrome metric HDMI camera.

Composite images of the dissected body parts were used as a reference to draw taxonomic figures as per Coleman (2003) using the open-source vector illustration software Inkscape v. 1.4.2. Length ratios for the appendages were derived from images using the open-source image analysis software ImageJ (Schneider et al. 2012). The holotype specimen of *Princaxelia malohi* sp. nov. is deposited at the Museum of New Zealand Te Papa Tongarewa (**NMNZ**) under the accession number CR.028062. The holotype specimen of *Princaxelia kahat* sp. nov. is deposited at the Smithsonian National Museum of Natural History (**USNM**) under the accession number of USNM 1775248. Additional material of both species is stored within the Minderoo-UWA Deep-Sea Research Centre collection at The University of Western Australia.

### Scanning electron microscopy

Supplemental SEM micrographs of selected diagnostic features from gnathopods of additional specimens from *Princaxelia malohi* sp. nov. were taken using a JEOL NeoScope JCM-7000 benchtop scanning electron microscope (SEM). Gnathopods were rinsed with 100% ethanol 4× and processed in a PELCO Biowave microwave processor for 1 min each rinse. Samples were subsequently dried using a Polaron E3000 critical point dryer as per the manufacturer instruction. Specimens were mounted on aluminium stubs with the lateral side facing up using conductive carbon tape and coated in carbon using an HHV Auto-306 Carbon Coater. Gnathopods were then coated with a layer of platinum using a Leica EM ACE600 Metal Coater. Images from the SEM were captured using secondary electron imagery with the machine set to high vacuum mode, and an accelerating voltage of 15.0 kV and working distance of 13.7–14.5 mm was used for all secondary images. Additional images were taken using backscattered electron imagery on a FEI Verios XHR SEM with an accelerating voltage of 20.0 kV and a working distance of 5.5 mm. All micrographs were generated at the Centre for Microscopy Characterisation and Analysis at The University of Western Australia.

### DNA barcoding

All five individuals of *Princaxelia malohi* sp. nov. and 12 individuals of *Princaxelia kahat* sp. nov. were selected for barcoding. All types specimens were included in the DNA barcoding pool to reduce future taxonomic issues (D’Acoz and Havermans 2015). Total genomic DNA was extracted from the pleopods using the DNeasy Blood and Tissue Kit (Qiagen) according to the manufacturer’s protocol. The mitochondrial barcoding regions, 16S rRNA, 28S rRNA, and cytochrome *c* oxidase subunit I (COI), were targeted based on sequences available in GenBank®. The primers used for 16S rRNA were from (Folmer et al. 1994), 6SFt_amp (5’-GCRGTATIYTRACYGTGCTAAGG) and 16SRt_amp2 (5’-CTGGCTTAAACCGRTYTGAACTC). COI was amplified with LCO1490 and HCO12198 (Folmer et al. 1994). PCR products were assessed using 96-well E-gels (Invitrogen) and purified with AMPure XP paramagnetic beads (Beckman Coulter). 28S was amplified using the primers from Foltz et al. (2007), 28Srtw (5’-ACTTTCCCTCAYGGTACTTGT) and 28Sftw (5’-AGAAACTAACMAGGATTCCYYTAGTA). Sequencing preparations used the Thermo Fisher Scientific Applied Biosystems BigDye Cycle Sequencing Kit, and clean-up was done using the CleanSEQ Dye-Terminator Removal Protocol (Beckman Coulter). Sequencing was performed on a 3730xl capillary sequencer (Thermo Fisher Scientific Applied Biosciences) at Macrogen (Korea). Sequence assembly and editing were done in Geneious Prime 2023.0.1 (Kearse et al. 2012). Newly generated sequences were deposited in GenBank® under the following accession numbers (PZ585448; PZ611770–PZ611786; Suppl. material 1).

Generated sequences were assembled and cleaned in Geneious Prime v. 2025.1.3. Newly sequenced data was further supplemented by all publicly available sequence data listed on GenBank® (Suppl. material 1). Individual gene datasets (COI and 16S rRNA) were aligned using the Geneious MAFFT alignment plugin (Katoh et al. 2005). All COI alignments were subsequently checked for stop codons prior to further analyses. Pairwise genetic distances were calculated separately for each marker using a custom Python implementation of uncorrected p-distance and Kimura two-parameter (K2P) distance. Sites containing gaps or ambiguous nucleotides were excluded using pairwise deletion.

## Systematics

### Order Amphipoda

**Family Pardaliscidae Boeck, 1871**

**Genus *Princaxelia* Dahl, 1959**

#### Princaxelia malohi

Taxon classificationAnimaliaAmphipodaPardaliscidae

Duffy & Wainwright sp. nov.

246DFB8B-7AFE-5602-8CBB-560DDAF9A584

https://zoobank.org/E268BA46-CB89-458F-92EF-234240557140

Figs 2–8

##### Material examined.

***Holotype***. • Mature ♂, body length 35.1 mm; Tonga Trench (23°29.238'S, 174°59.890'W); 8,200 m; 18 July 2024; Baited lander. NMNZ accession number: CR.028062, GenBank® accession number CO1: PZ585448; 16S: PZ611777; 28S: PZ611786.

##### Other material.

• 2 ♂, collection data same as holotype. • 1 ♂, 20.0 mm; Tonga Trench (23°29.238'S, 174°59.890'W); 8,200 m; 18 July 2024; baited lander. • Juvenile, 16.6 mm; Tonga Trench (23°28.806'S, 174°59.906'W), 8,350 m; 18 July 2024; baited lander. GenBank® accession numbers 16S: PZ611774–PZ611776; 28S: PZ611783–PZ611785.

##### Diagnosis.

Primary flagellum article 1 of male antennae 1 elongate. Accessory flagellum of male antennae 1 article 1 enlarged and flattened, with two small articles at distal end. Maxilla 1 inner plate with two plumose setae; palp article 2 expanded with 11 or 12 apical robust setae. Maxilla 2 outer plate with more than three plumose setae. Maxilliped inner plate with two apical setae. Dactylus of gnathopods 1 and 2 with a single strong projection on posterior margin near the base. Dorsal margin of coxa 5 straight. Ventral margin of coxa 7 shallowly concave. Telson lobes uniformly tapering distally with one apical spine per lobe.

##### Description based on holotype, male.

***Head***. (Fig. 4A–C) Small, similar to length of pereonite 1; rostrum very short, slightly pointed; lateral cephalic lobe prominent; corner rounded, angled distally upward; eyes absent. ***Antennae 1*** peduncle articles 1–3 with length ratio 1.0: 0.64: 0.35; peduncular article one broadened, with cluster of setae on ventral margin, some weakly plumose; posterior of peduncular articles 2 and 3 with short setae clusters. Primary flagellum article one thin and elongate with length 4× width; dorsal submargin with large clusters of thin sensory setae (aesthetascs), forming a callynophore. Accessory flagellum 3-articulate; article 1 elongate and flattened with setae on posterodorsal margin; articles 2 and 3 very short, about five percent of accessory flagellum. Primary flagella broken at article 19. ***Antennae 2*** length 0.4× body length. Posterior margin of peduncular articles 1 and 2 with short setae clusters; peduncular article 3 slightly longer than article 4. Flagellum with 36 articles.

***Mouthparts***. (Fig. 4D–O) ***Upper lip*** medial face asetose, with asymmetrical setose ventral margin. ***Mandible*** lacinia mobilis present on left and right side, highly asymmetric, left lacinia mobilis broad and multi-dentate, right lacinia mobilis thin with broad projection at proximal end; setal row with 21 robust setae Mandible incisors with strong posterodistal projection, slightly asymmetric; molar absent. Mandible palp 3-articulate, article 1 asetose, short; article 2 arched medially with 12 setae on medial margin; article 3 0.85× length of article 2, with 17 setae across all margins. ***Lower lip*** setose with distinct outer lobes present; mandibular process absent or severely reduced. ***Maxilla 1*** inner plate small with two apical plumose setae; outer plate arched medially with nine robust apical setae, one large claw-like projection at the distal end. Maxilla 1 palp 2-articulate; article 1 subrectangular, setae on posterior; article 2 expanded distally with 13 apical and eight marginal robust setae, apical submargin with setae. ***Maxilla 2*** inner plate with 15 plumose setae along apical to medial margin, outer plate slightly longer and thinner than inner plate with four plumose setae along the apical margin and two along lateral margin. ***Maxilliped*** with inner and outer plates and palp; inner plate small and subtriangular with two apical plumose setae; outer plate rounded, reaching base of palp article 2, with 29 robust setae along medial to apical margin. Maxilliped palp long, 4-articulate; article 1 short, lateral margin with sparse setae, medial margin with dense setae; article 2 longest article, similar pattern of setae as article 1; article 3 long, slightly shorter than article 2 with similar setae pattern; article 4 slender with four strong claw-like projections on medial margin, tapered distally.

**Figure 2.**
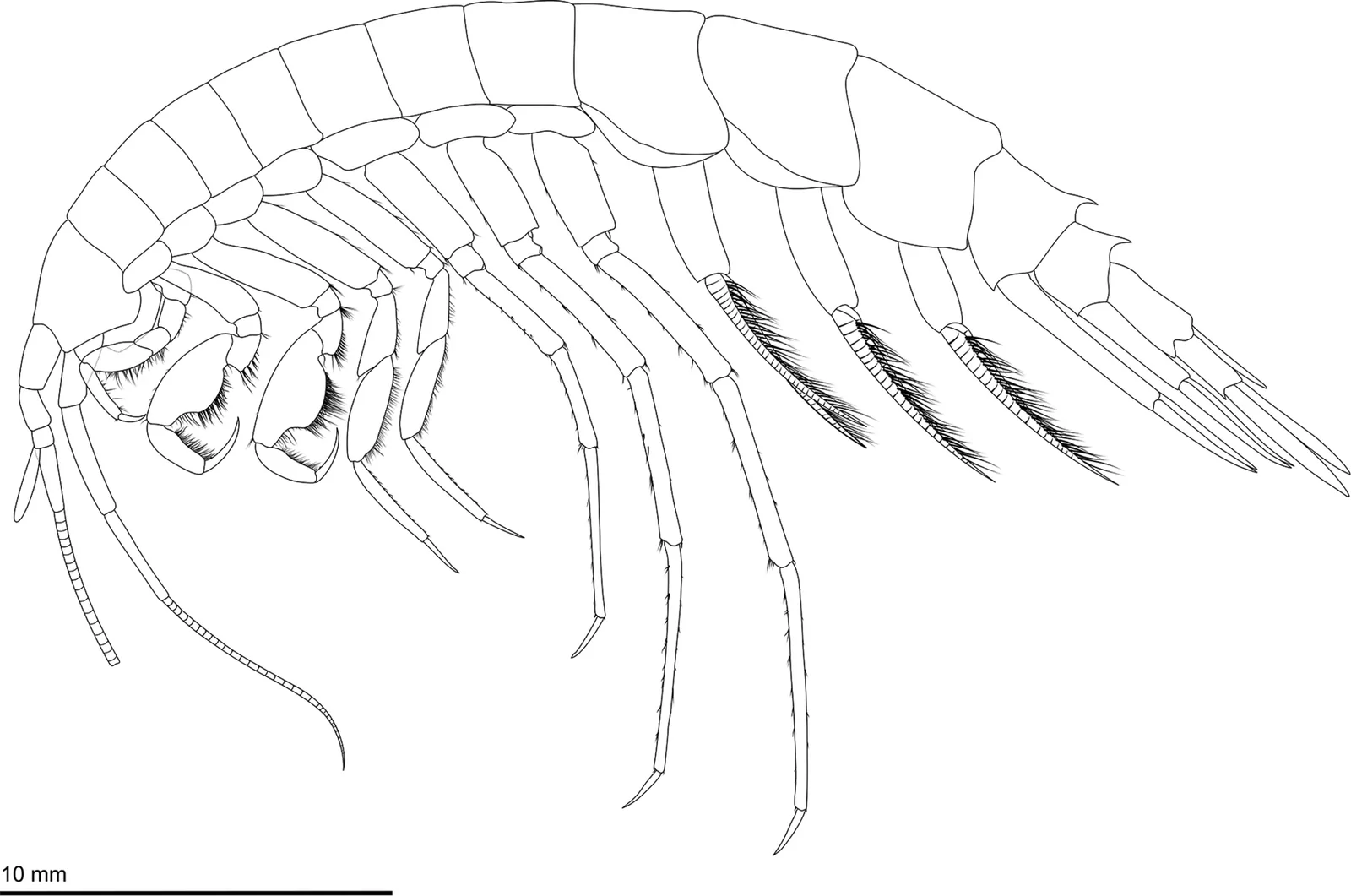
*Princaxelia malohi* sp. nov., holotype ♂ (body length 35.1 mm). Habitus, lateral view.

**Figure 3.**
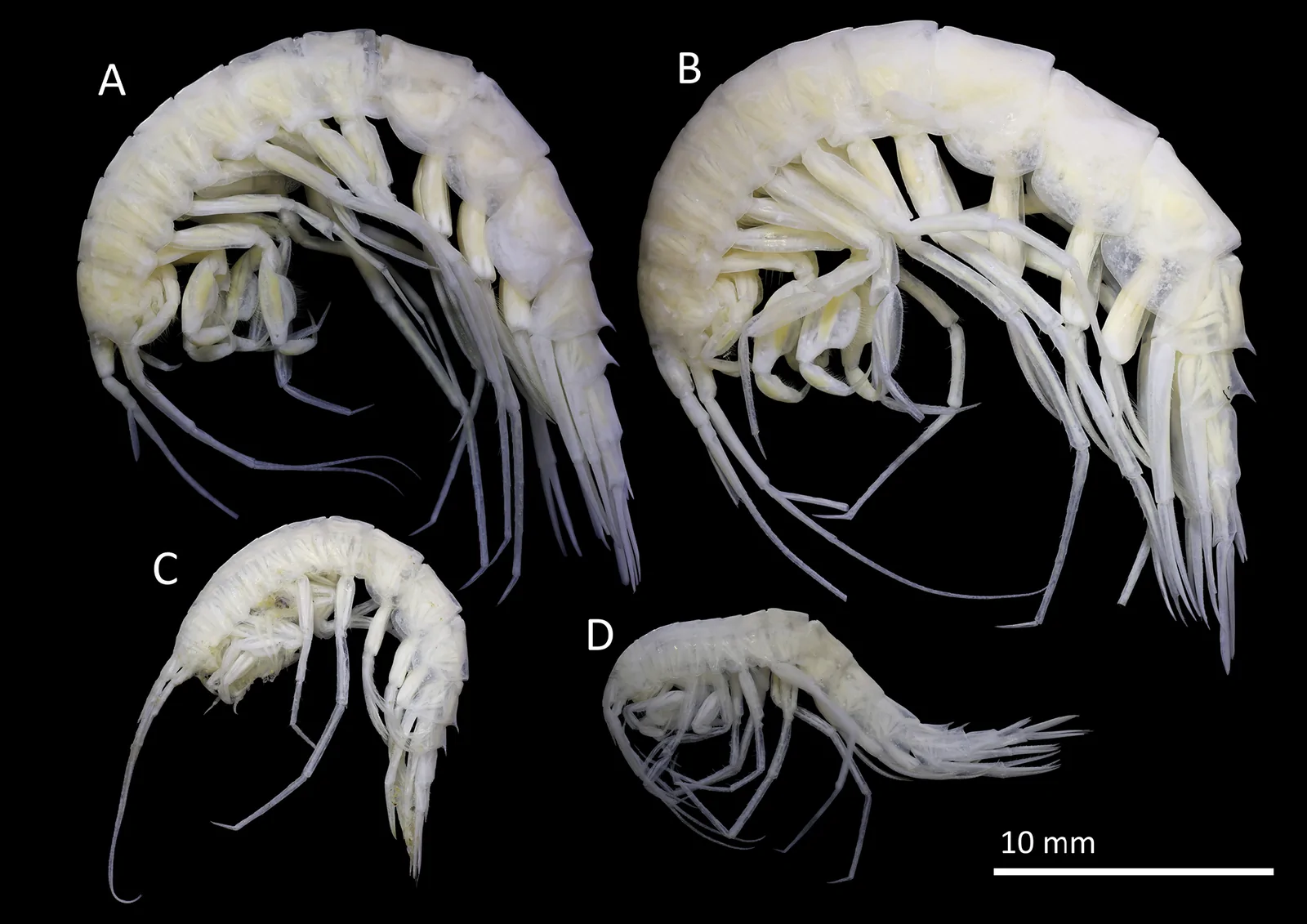
*Princaxelia malohi* sp. nov. **A**. Holotype ♂, 35.1 mm; **B**. Adult ♂, 39.5 mm; **C**. Adult ♂, 20.0 mm; **D**. Juvenile, 16.6 mm.

***Body***. (Figs 2, 3) Pereon with subrectangular pereonites, dorsal and ventral margin smooth; pleon with dorsal surface smooth; dorsal margin of urosomites 1 and 2 with single thorn-like projection each, tapered distally, urosomite 3 dorsal margin smooth. Coxal gills present on gnathopod 2, pereopods 3–6; gills 2–4 elongate, gills 5 and 6 short and ovular.

**Figure 4.**
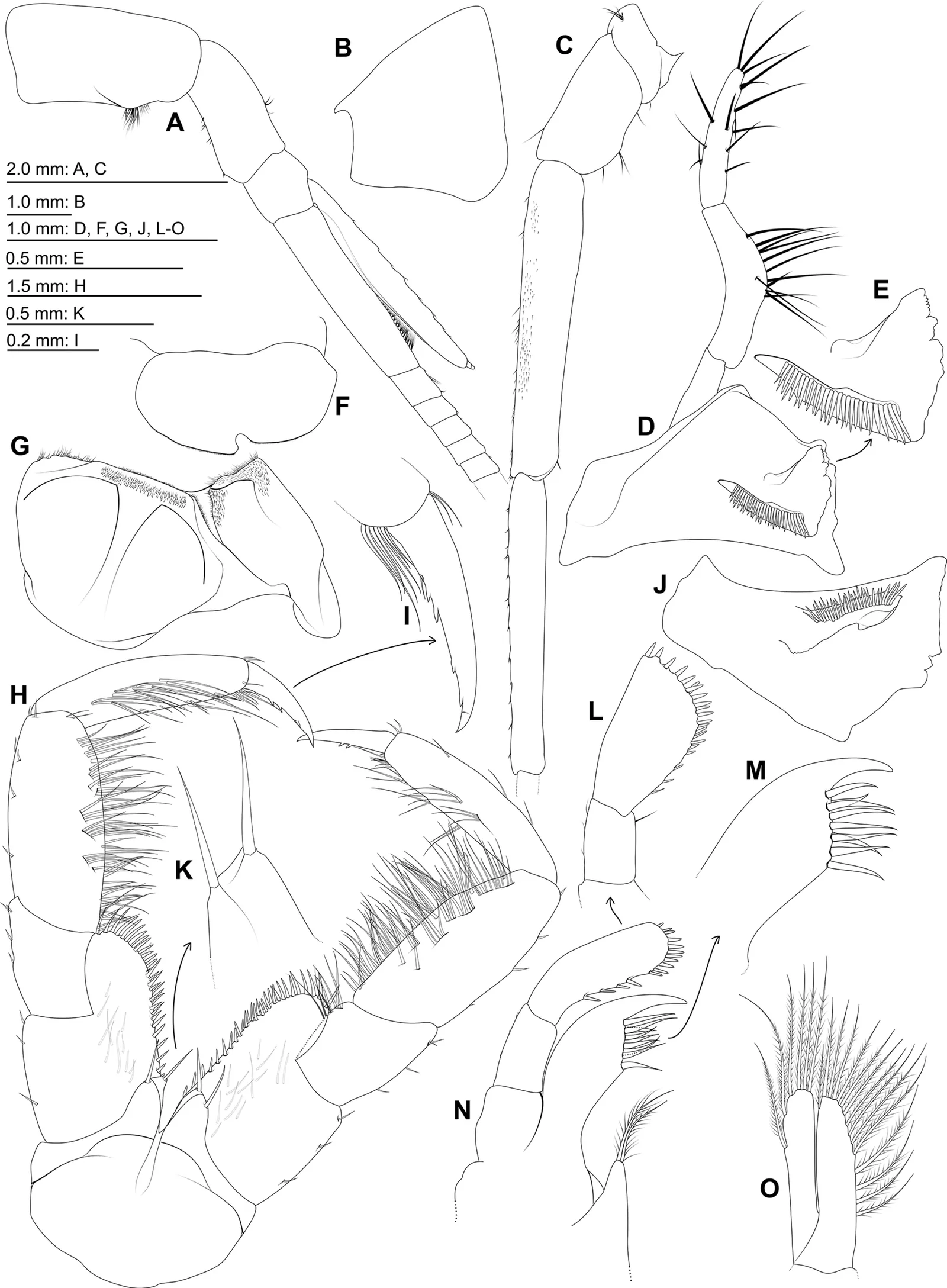
*Princaxelia malohi* sp. nov., holotype ♂. **A**. Antenna 1, left side, lateral, some primary flagella articles omitted; **B**. Head, lateral; **C**. Antenna 2, lateral, flagella omitted; **D**. Left mandible, dorsal; **E**. Left mandible lacinia mobilis, dorsal; **F**. Upper lip; **G**. Lower lip, dorsal, left lobe omitted; **H**. Maxilliped, dorsal; **I**. Maxilliped palp article 4; dorsal; **J**. Right mandible, dorsal, palp omitted; **K**. Maxilliped inner plate, dorsal; **L**. Maxilla 1 palp, lateral; **M**. Maxilla 1 inner plate, lateral; **N**. Maxilla 1, dorsal; **O**. Maxilla 2, dorsal.

**Figure 5.**
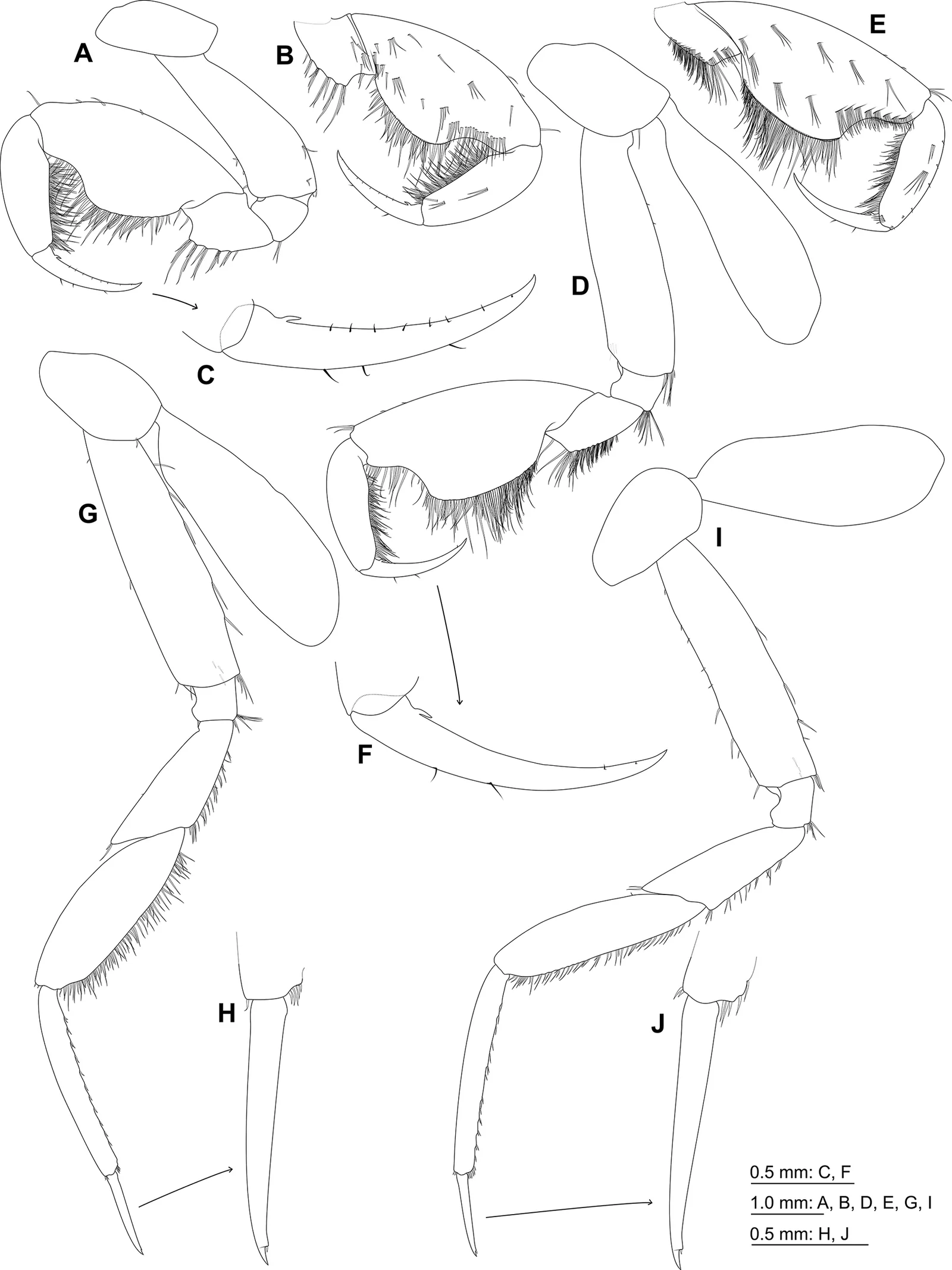
*Princaxelia malohi* sp. nov., holotype ♂. **A**. Gnathopod 1, lateral; **B**. Gnathopod 1, medial, basis and ischium omitted; **C**. Gnathopod 1 dactylus, lateral; **D**. Gnathopod 2 with coxal gill, lateral; **E**. Gnathopod 2, medial, basis and ischium omitted; **F**. Gnathopod 2 dactylus, lateral; **G**. Pereopod 3 with coxal gill, lateral; **H**. Pereopod 3 dactylus; lateral; **I**. Pereopod 4 with coxal gill, lateral; **J**. Pereopod 4 dactylus, lateral.

**Figure 6.**
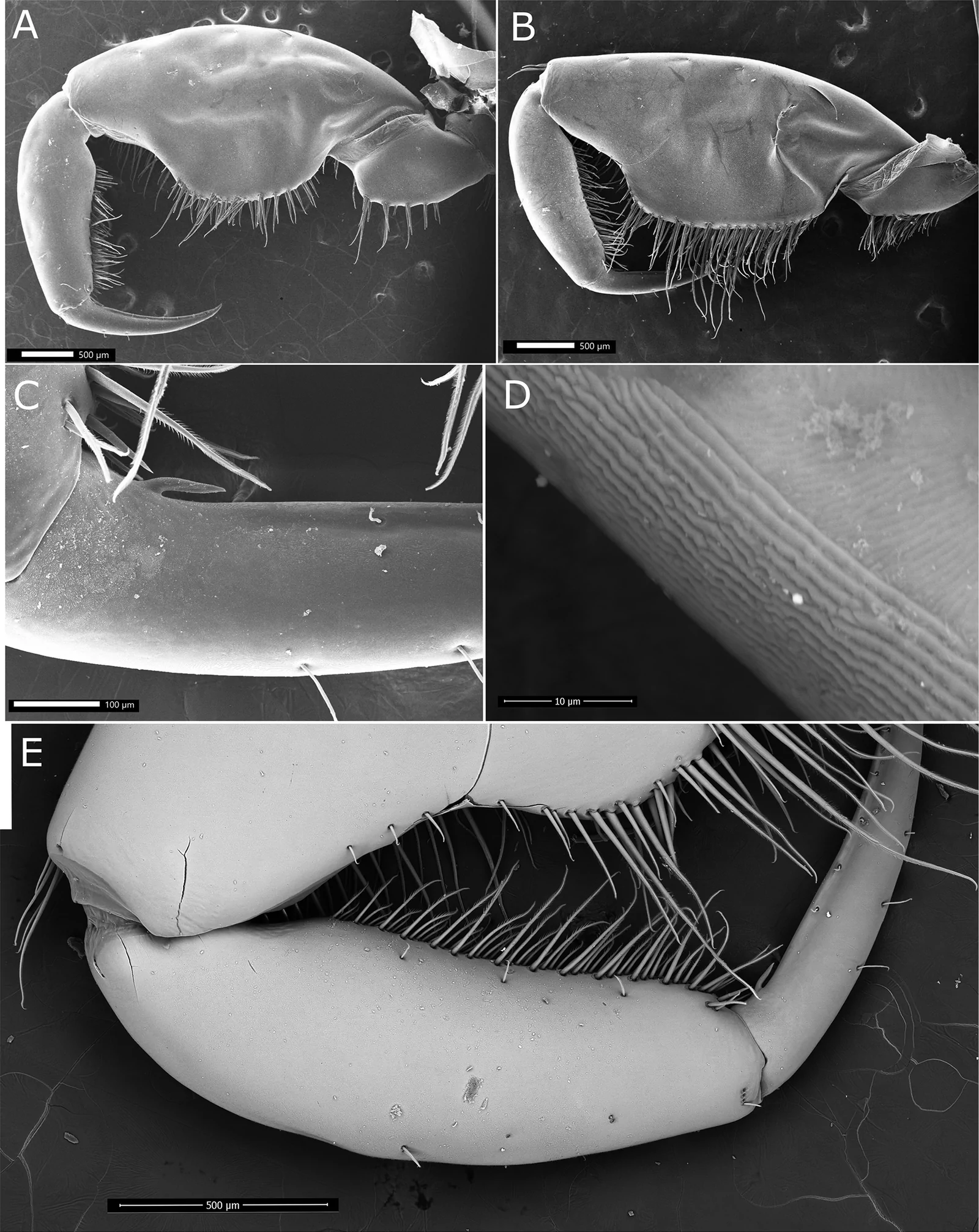
SEM micrographs of *Princaxelia malohi* sp. nov., adult ♂, 39.5 mm. **A**. Gnathopod 1, lateral; **B–E**. *Princaxelia malohi* sp. nov., adult ♂, 33.5 mm; **B**. Gnathopod 2, lateral; **C**. Gnathopod 2 dactylus projection, lateral; **D**. Detailing of surface texture on ventral margin of gnathopod 2 dactylus; **E**. Backscattered composite image of gnathopod 2 propodus, lateral.

***Gnathopods***. (Figs 5A–F, 6) ***Gnathopod 1*** coxa subrectangular and slightly bilobate, ventral margin straight, posterior submargin with setae; basis slightly arched distally, with sparse setae on medial face, length 2.8× width; ischium small and subquadrate, single clump of setae near base of merus on posterior margin; merus broad, concave along proximal margin, ventral margin with evenly separated clumps of setae; carpus deep and rounded, length 2× width, dorsal margin smooth with setae sparse, lateral face asetose, posterior ventral submargin and medial face with dense clumps of setae; propodus narrower than carpus, posterior margin with dense setae; dactylus thin, dorsal and ventral margin with setae, posterior margin with one strong projection near base. ***Gnathopod 2*** coxa subrectangular and slightly bilobate, ventral submargin with sparse setae; basis straight and elongate, length 3.8× width, sparse setae on posterior margin; ischium similar to gnathopod 1 with denser posterior setae clump; merus more elongate than gnathopod 1, numerous setae clumps on ventral submargin; carpus deep, subrectangular, length 2.2× width, dorsal margin smooth with sparse setae, lateral face asetose, ventral submargin and medial face densely setose; propodus similar to gnathopod 1, with the exception of having barbed robust setae (can be seen on the topmost setae of Fig. 6C); dactylus with majority of posterior margin asetose, otherwise similar to gnathopod 1.

**Figure 7.**
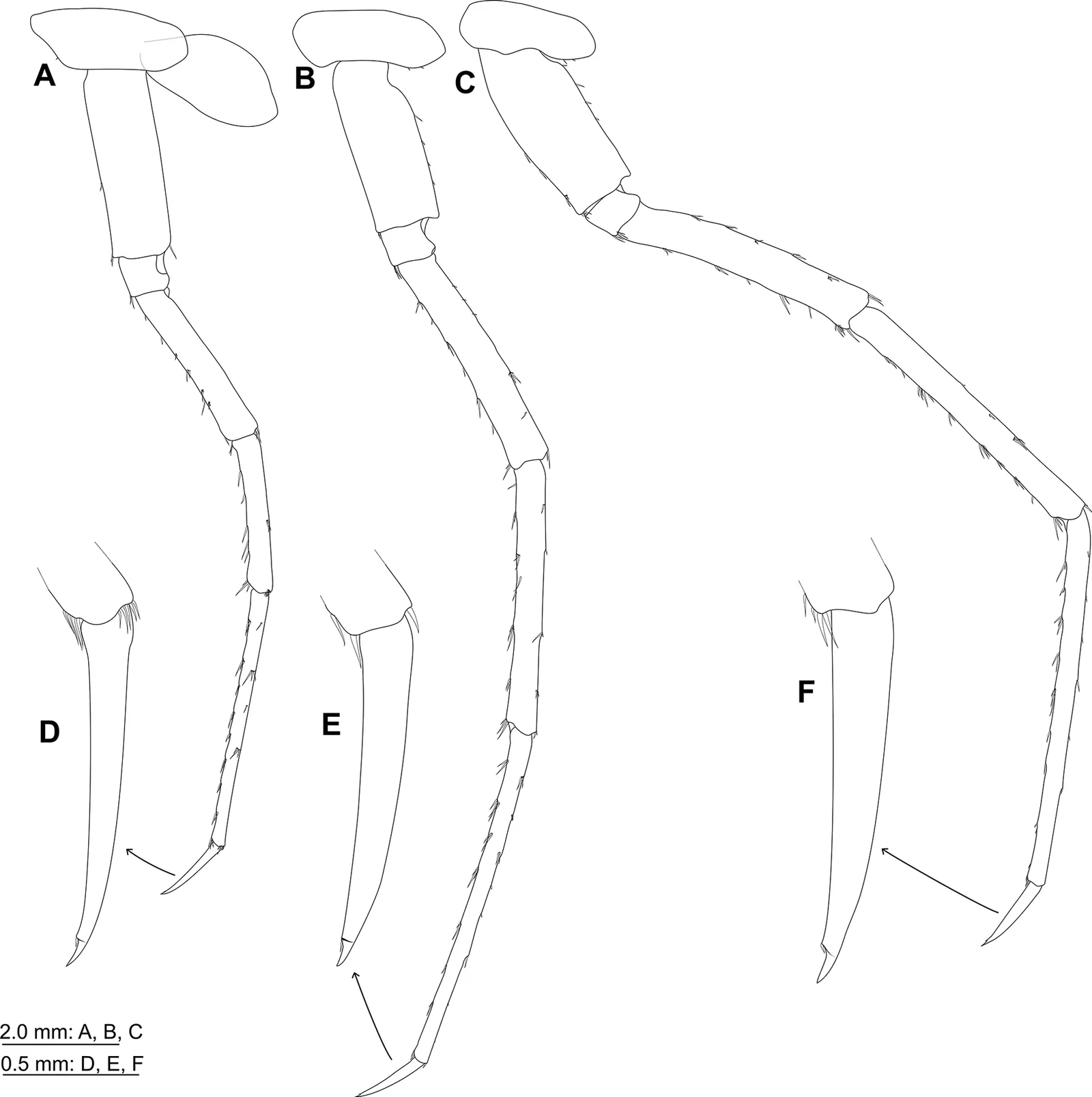
*Princaxelia malohi* sp. nov., holotype ♂. **A**. Pereopod 5 with coxal gill, lateral; **B**. Pereopod 6, lateral; **C**. Pereopod 7, lateral; **D**. Pereopod 5 dactylus, lateral; **E**. Pereopod 6 dactylus, lateral; **F**. Pereopod 7 dactylus; lateral.

***Pereopods***. (Figs 5G–J, 7) ***Pereopod 3*** coxa ovular, slightly bilobate along dorsal margin, ventral submargin rounded with sparse setae; basis elongate, anterior and posterior margin setose, large clump of setae on posterodistal angle; ischium anterior margin concave, dense clump of setae on posterodistal margin; merus, carpus, propodus, and dactylus length ratio 1.0: 1.22: 1.19: 0.52; merus elongate, distal margin tapered, dorsal and ventral margins straight, posterior margin setose; carpus elongate, rounded; dorsal margin asetose, posterior margin with dense setae. Propodus elongate, thin, anterior margin asetose, posterior margin with short setae; dactylus slender with single robust seta near distal end. ***Pereopod 4*** coxa ovular, not bilobate, slightly tapered proximally, asetose; basis, ischium, merus, carpus, and dactylus shape and setae arrangement similar to pereopod 3. ***Pereopod 5*** coxa subrectangular, tapered near proximal end, dorsal margin slightly convex, ventral margin straight, sparsely setose, length 2.6× width; basis broad and rectangular, length 2.9× width, posterior margin with many thin setae, anterior margin sparsely setose, posterodistal corner rounded; ischium short and rectangular, anterodistal corner with clump of setae, anterior margin slightly concave; merus, carpus, propodus, and dactylus length ratio 1.0: 0.89: 1.47: 0.43; merus thin, anterior and posterior margin setose; medial face setose; carpus slightly shorter than merus, with similar setae arrangement; propodus elongate, 60% longer than carpus, setae along anterior submargin and posterior margin; dactylus slender, one robust seta near the distal end. ***Pereopod 6*** 1.28× longer than pereopod 5; coxa subrectangular, length 2.8× width, slightly tapered at distal end, ventral margin slightly concave, distal end sparsely setose; entire pereopod long and slender, 1.28× longer than pereopod 5; basis broad, length 2.4× width, posterodistal corner quadrate; ischium similar to pereopod 5; merus, carpus, propodus, and dactylus similar to pereopod 5, length ratio 1.0: 1.16: 1.46: 0.31. ***Pereopod 7*** 1.04× longer than pereopod 6; coxa slender, bilobate, rounded at distal end ventral margin slightly concave, posterodistal margin with sparse setae, medial margin with penile papillae; penial papillae large, rounded and faced medially; basis slightly broader than pereopod 6, length 2.11× width; ischium, merus, carpus, propodus, and dactylus similar to pereopod 6.

**Figure 8.**
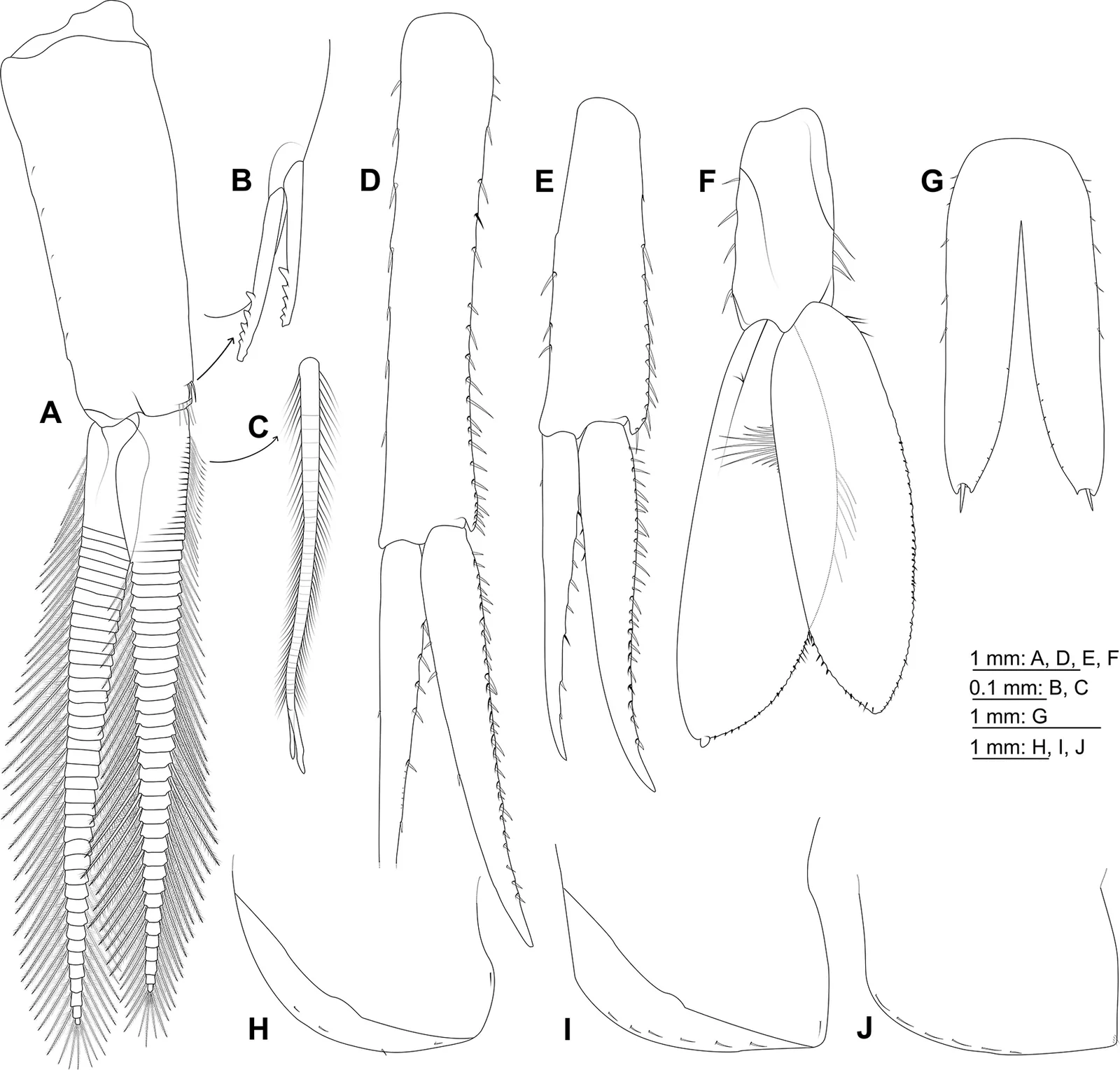
*Princaxelia malohi* sp. nov., holotype ♂. **A**. Pleopod 2, posterior; **B**. Coupling spines (retinacula) on pleopod; **C**. Bifurcate (clothespin) setae on outer lobe of pleopod; **D**. Uropod 1, dorsal; outer ramus broken distally; **E**. Uropod 2, dorsal; **F**. Uropod 3, dorsal, most rami setae broken or missing; **G**. Telson, dorsal; **H**. Epimeron 1, lateral; **I**. Epimeron 2, lateral; **J**. Epimeron 3, lateral.

***Pleon and urosome***. (Fig. 8) ***Pleopods*** with paired coupling setae (retinacula) on medial distal margin of peduncle; coupling setae with five hooks on inner margin; rami of similar length; inner ramus with numerous bifid (clothespin) setae on inner basal margin; ramus articles wide, with dense plumose setae along both side margins, article at distal end with additional plumose setae on ventral margin. ***Epimeron*** setose on ventral submargin; ***epimeral plate 1*** ventral margin curved, with strong groove above the ventral margin, posterodistal corner rounded; ***epimeral plate 2*** subrectangular with strong groove above the ventral margin, posterodistal corner subquadrate; ***epimeral plate 3*** subrectangular, posterodistal corner quadrate. ***Uropod 1*** peduncle longer than rami, seven basofacial setae, distomedial projection weak; inner ramus 0.8× length of peduncle, medial margin setose, sparse setae on lateral margin; outer ramus damaged near the distal end, medial margin setose, lateral margin with few setae. ***Uropod 2*** peduncle about same length as rami, three basofacial setae, distomedial peduncular projection similar to uropod 1; rami with setae along medial margins, sparse setae on lateral margin; inner ramus 1.1× length of outer ramus. ***Uropod 3*** peduncle short and subrectangular, length 2.2× width, medial and lateral margins setose; rami paddle-like, wide, and ovular; inner ramus with setae on all margins; outer ramus with very short setae along medial margin, a single large bump on distal end. Telson length 2.5× width, cleft extending 78% of length, lobes weakly tapered distally with many setae, apex of each lobe concave, with one robust seta each.

##### Variations.

The maxilla 2 inner plate setae number varies with specimen size. One specimen (BL 33.5 mm, ♂) has four apical setae on the right maxilla 2 inner plate and three on the left. The robust setae of the outer plate of the maxilla 1 vary from seven to nine. Article 3 of the maxilliped with numbers of projections ranging from three to five.

##### Supplementary material.

*In situ* video footage was also captured from the baited landers dropped in the Tonga Trench (https://doi.org/10.5281/zenodo.20756281), which provides further insight on the locomotion and predation habits of *Princaxelia*.

##### Differential diagnosis.

*Princaxelia malohi* sp. nov. is the only member of *Princaxelia* to have two plumose setae on the inner plate of the maxilla 1 and consistently more than three plumose setae on the outer plate of the maxilla 2. It is also the only reported member of the genus to have a single basal projection on the dactylus of both gnathopods.

##### Etymology.

The specific epithet *malohi* is derived from the Tongan word for mighty or powerful. This refers to back to the status of this animal as a predator, where it uses its strength to subdue prey, as well as the adaptations it must have to survive in hadal depths. It is treated as an indeclinable noun in apposition.

#### Princaxelia kahat

Taxon classificationAnimaliaAmphipodaPardaliscidae

Duffy & Wainwright sp. nov.

F10BB474-9B6B-589E-A118-2BDFD0945C5F

https://zoobank.org/1FEADE39-34F2-4EF4-BDF3-12288BD283F7

**Figure 9.**
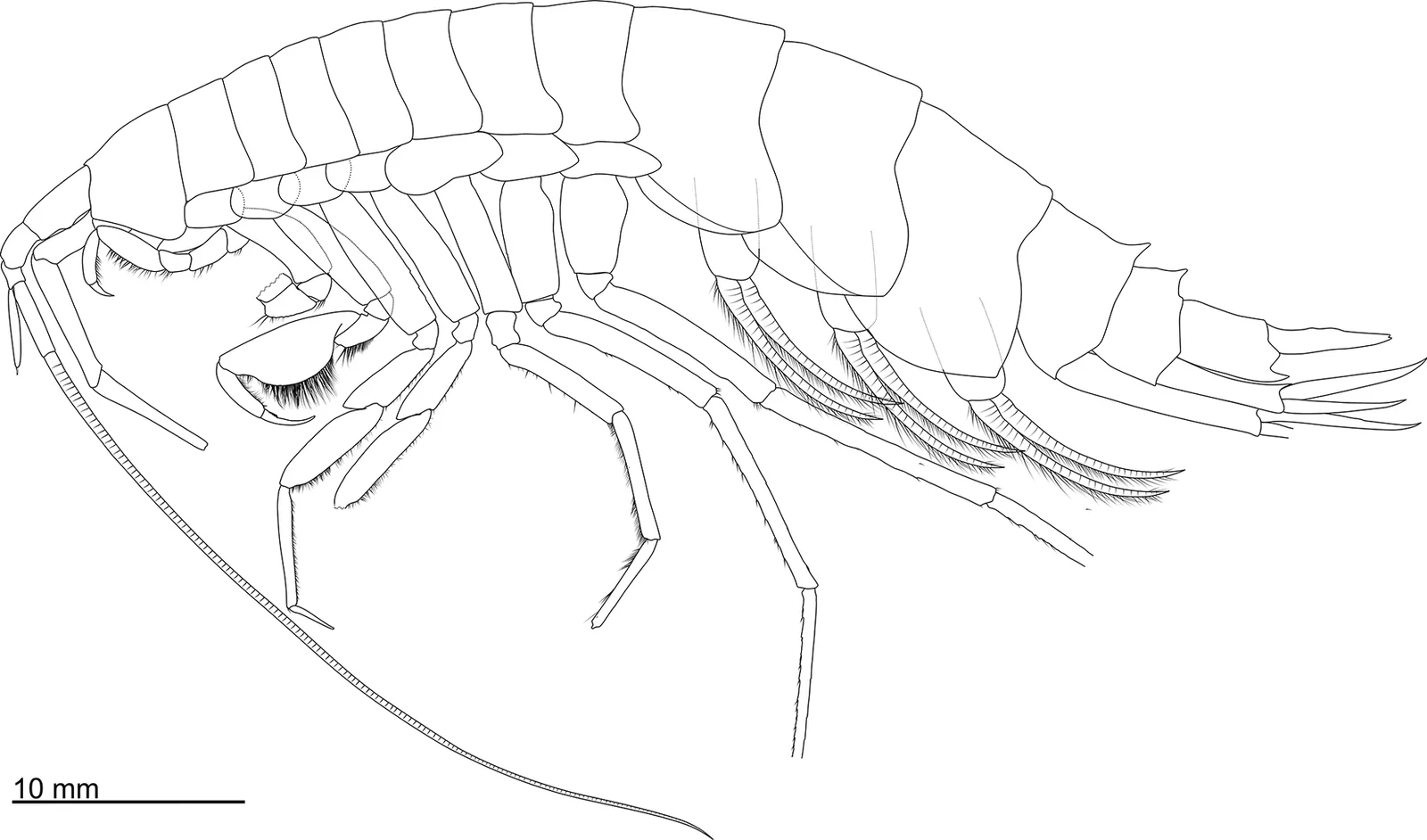
*Princaxelia kahat* sp. nov., holotype ♂ (59.3 mm). Habitus, lateral view.

**Figure 10.**
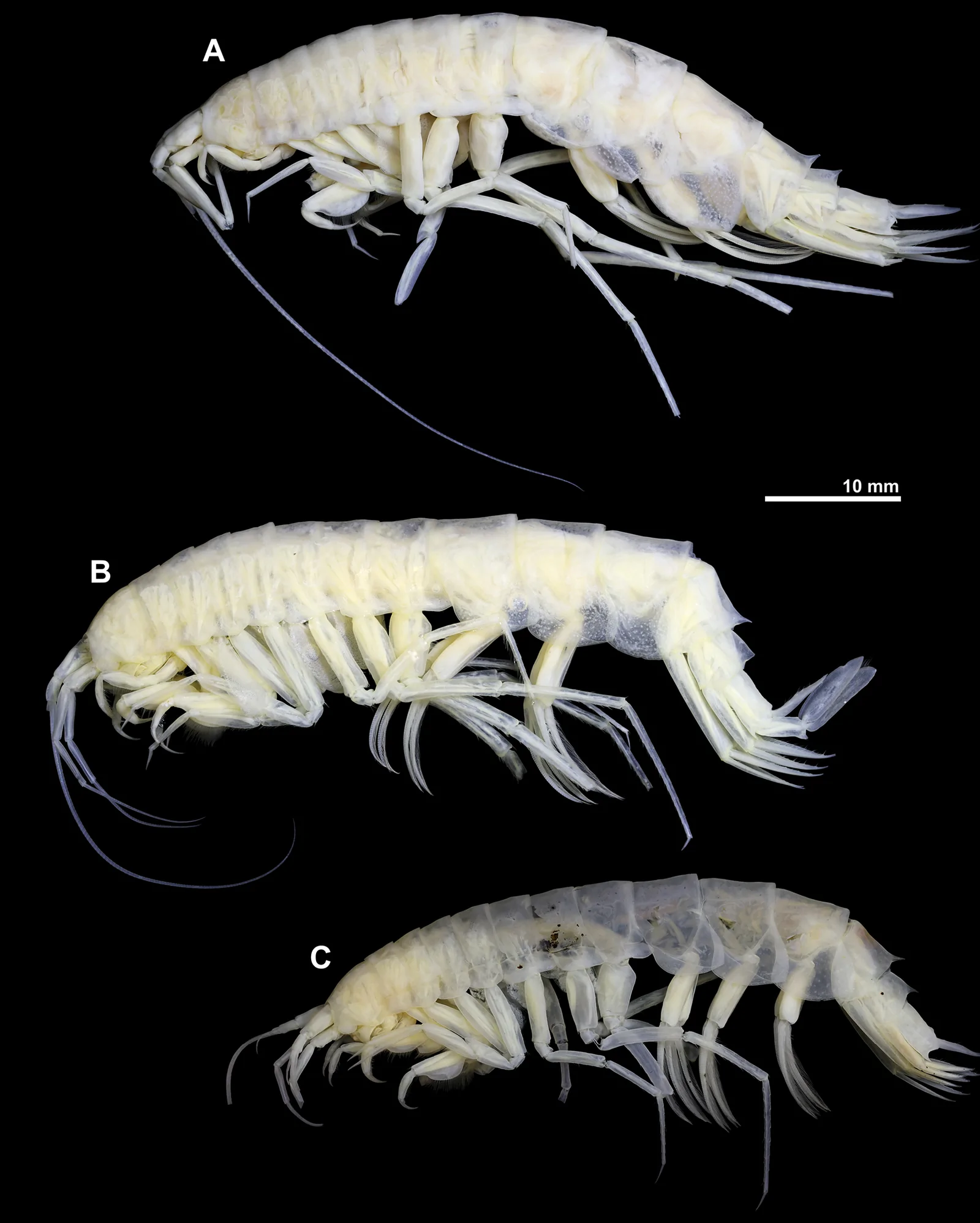
*Princaxelia kahat* sp. nov. **A**. Holotype ♂, 59.3 mm; **B**. Adult ♀, 61.8 mm; **C**. Adult ♀, 53.4 mm.

**Figure 11.**
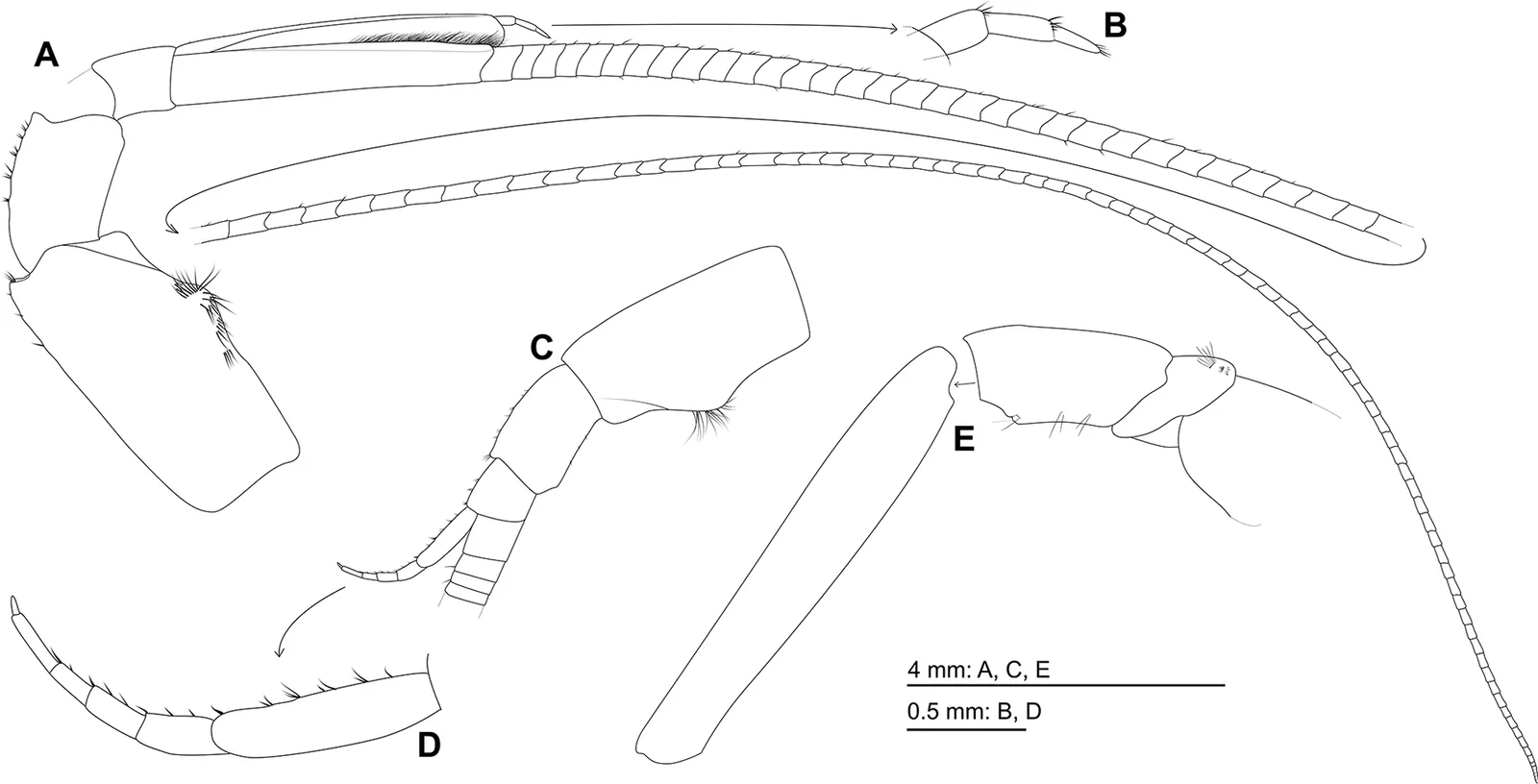
*Princaxelia kahat* sp. nov. **A**. Holotype ♂ right antennae 1, lateral; **B**. Holotype ♂ accessory flagellum article 2–4; **C**. Adult ♀ left antennae 1 lateral, distal articles of primary flagellum omitted; **D**. Adult ♀ antenna 1 accessory flagella; **E**. Holotype ♂ left antennae 2 lateral, article 5 and flagella missing.

**Figure 12.**
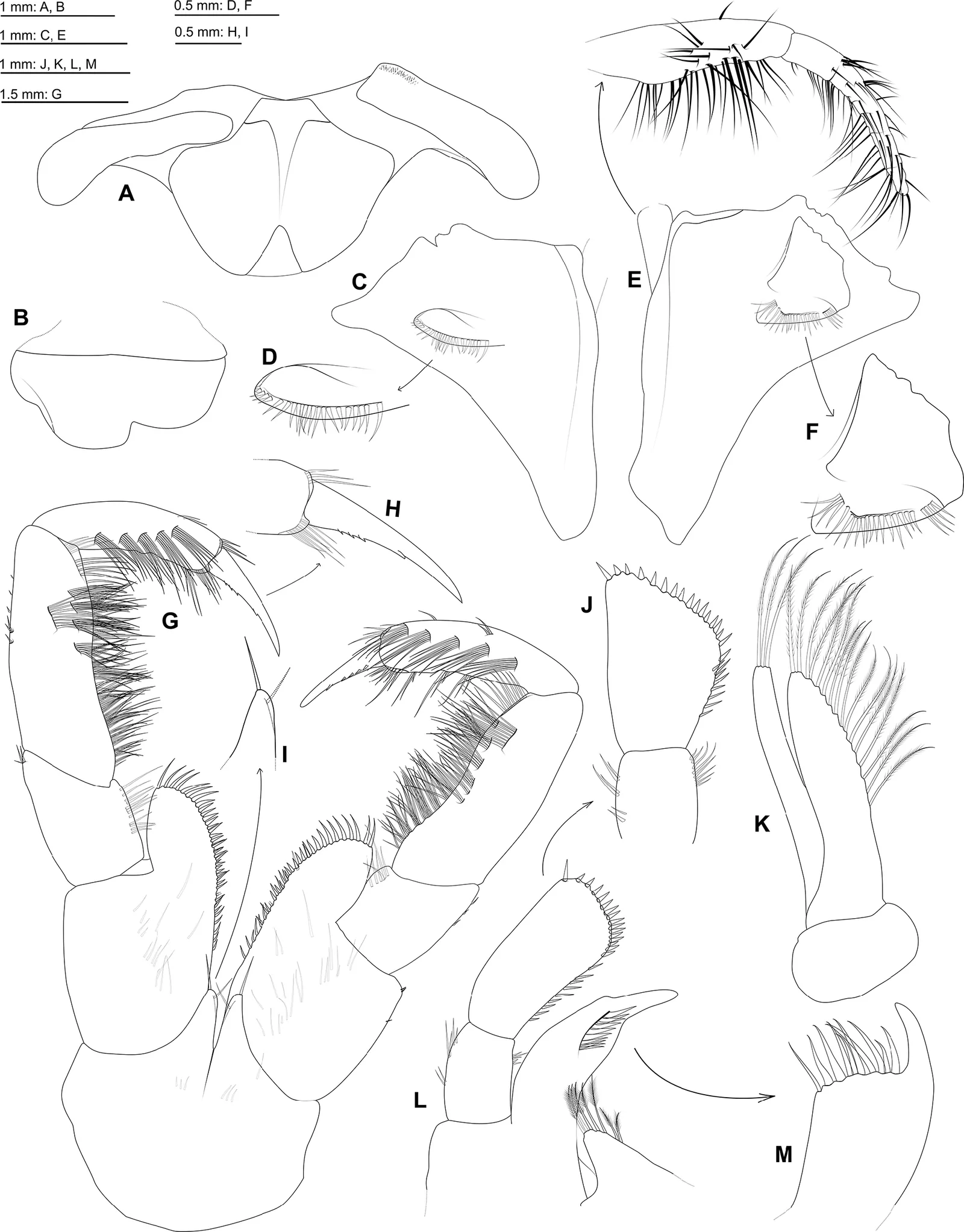
*Princaxelia kahat* sp. nov., holotype ♂. **A**. Lower lip, ventral; **B**. Upper lip; **C**. Right mandible, dorsal, palp omitted; **D**. Right mandible lacinia mobilis; **E**. Left mandible, dorsal; **F**. Left mandible lacinia mobilis; **G**. Maxilliped, dorsal; **H**. Maxilliped palp article 4; **I**. Maxilliped inner plate, dorsal; **J**. Maxilla 1 palp, medial; **K**. Maxilla 2, dorsal; **L**. Maxilla 1, ventral; **M**. Maxilla 1 outer plate, lateral.

**Figure 13.**
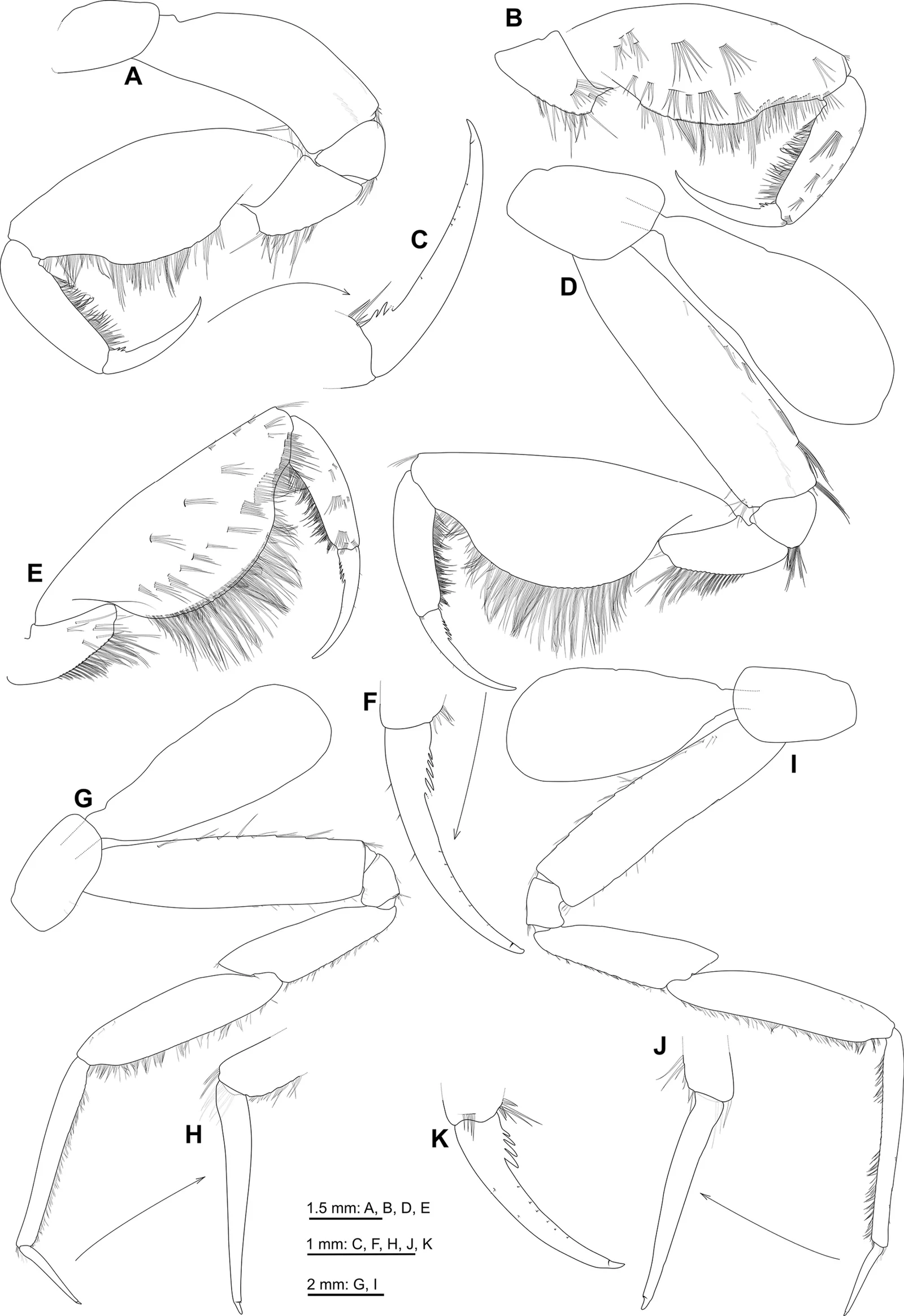
*Princaxelia kahat* sp. nov., holotype ♂. **A**. Gnathopod 1, lateral, coxa broken proximally; **B**. Gnathopod 1, medial, basis and ischium omitted; **C**. Gnathopod 1 dactylus, lateral; **D**. Gnathopod 2, lateral; **E**. Gnathopod 2, medial, basis and ischium omitted; **F**. Gnathopod 2 dactylus, lateral; **G**. Pereopod 3, lateral; **H**. Pereopod 3 dactylus, lateral; **I**. Pereopod 4, right lateral; **J**. Pereopod 4 dactylus, lateral; **K**. Adult ♀ 61.0 mm, dactylus of gnathopod 1, lateral.

**Figure 14.**
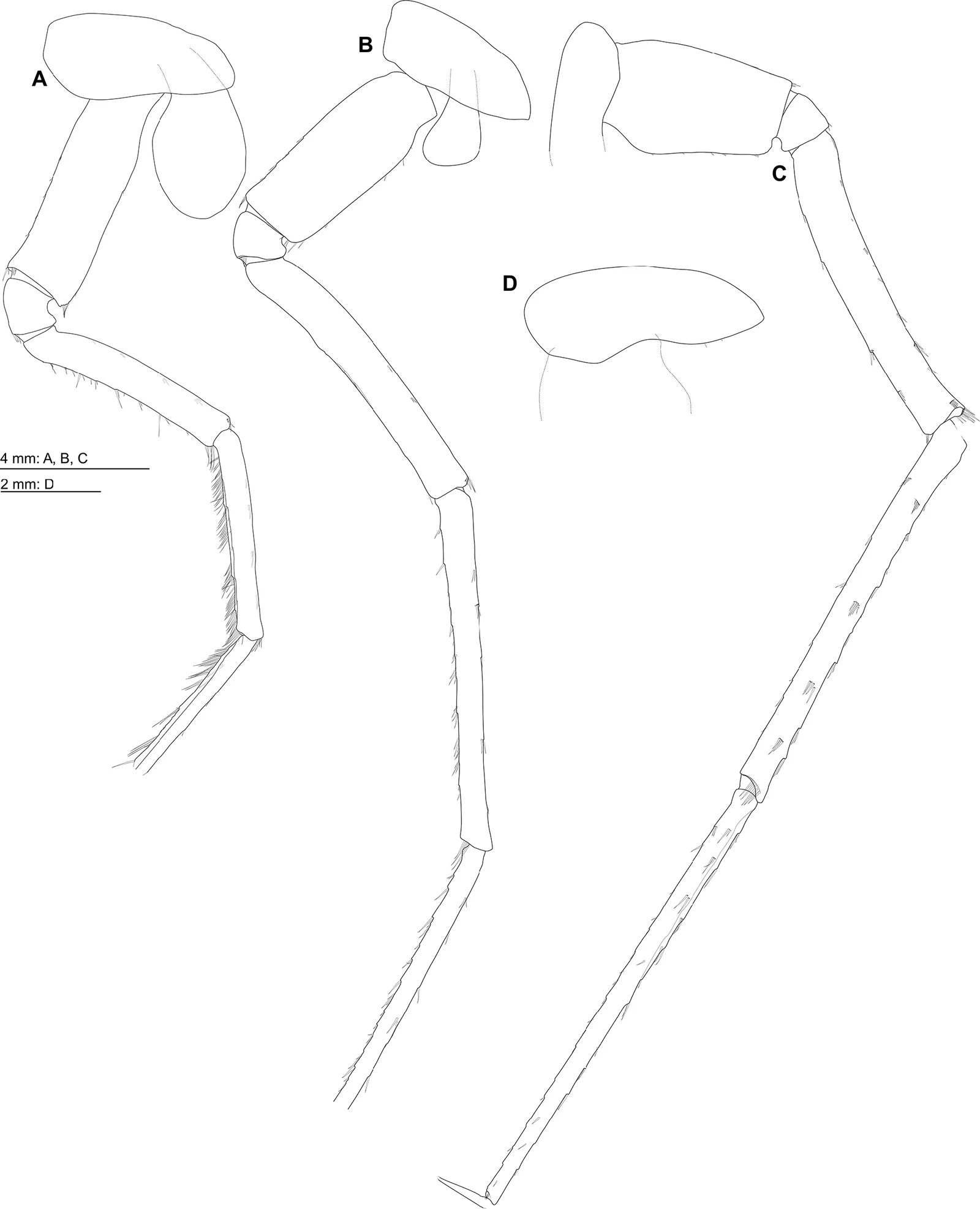
*Princaxelia kahat* sp. nov., holotype ♂. **A**. Pereopod 5, lateral, distal portion of propodus broken; **B**. Pereopod 6, lateral, distal portion of propodus broken; **C**. Pereopod 7, right lateral, coxa broken distally; **D**. Pereopod 7 coxae, left lateral.

**Figure 15.**
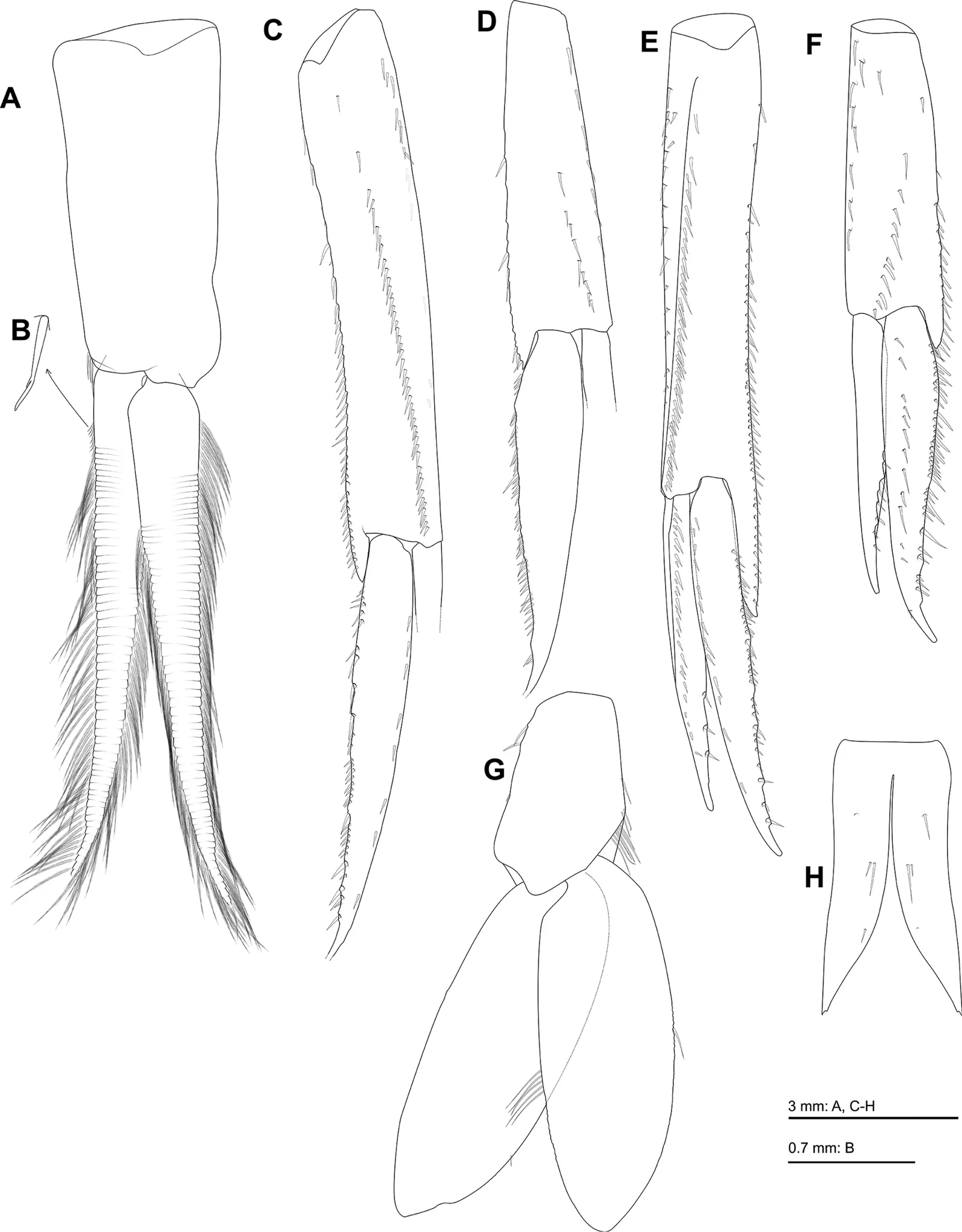
*Princaxelia kahat* sp. nov. **A–D**. Holotype ♂; **A**. Pleopod 2, lateral; **B**. Bifurcate seta on pleopod; **C**. Uropod 1, right dorsal, outer ramus broken distally; **D**. Uropod 2, right dorsal, outer ramus broken distally; **E–H**: Adult ♀ 61.0 mm; **E**. Uropod 1, left dorsal; **F**. Uropod 2, left dorsal; **G**. Uropod 3, dorsal, most rami setae broken or missing; **H**. Telson, dorsal.

Figs 9, 10, 11, 12, 13, 14, 15

Princaxelia magna : Jamieson and Weston 2023: table 2.

##### Material examined.

***Holotype***. • Mature ♂, body length 59.3 mm; Mariana Trench (12°17.316'N, 144°40.112'E); 8,143 m; 5 Dec. 2014; Baited lander. USNM accession number USNM 1775248. GenBank® accession number: 16S: PZ611773; 28S: PZ611782.

##### Other material.

• 4 ♂, 2 ♀; Mariana Trench (11°10.002'N, 142°30.972'E); 8,000 m; 26 Jan. 2017; Baited lander. GenBank® accession numbers: 16S: PZ611770–PZ611772; 28S: PZ611778–PZ611781.

##### Diagnosis.

Primary flagellum article 1 of male antennae 1 elongate and longer than half the body. Accessory flagellum of male antennae 1 article 1 enlarged and flattened, with three small articles at distal end. Upper lip slightly asymmetrical. Maxilla 1 inner plate with six plumose setae; palp article 2 expanded with 16 apical robust setae. Maxilla 2 outer plate with three plumose setae. Maxilliped inner plate with one apical plumose seta. Dactylus of gnathopods 1 with three and gnathopod 2 with five strong projections on the posterior margin near the base. Dorsal margin of coxa 5 slightly lower on distal end. Ventral margin of coxa 7 shallowly concave. Telson lobes broad and tapering distally at last 1/3.

##### Description primarily based on holotype, male.

***Head***. (Figs 9, 10) Slightly shorter than length of pereonites 1 and 2 combined; rostrum very short, slightly pointed; lateral cephalic lobe prominent; corner rounded; eyes absent.

***Antennae***. (Fig. 11) Extremely elongate, over 70 percent body length. Peduncle articles 1–3 with length ratio 1.0: 0.5: 0.2; peduncular article one broadened, with several clusters of setae arranged in rows on ventral margin, some weakly plumose, sparse setae on dorsal margin; posterior of peduncular article 2 with short setae clusters. Primary flagellum article one thin and elongate with length 7× width, dorsal submargin with dense clusters of thin sensory setae (aesthetascs), forming a callynophore. Primary flagella with 108 articles. Accessory flagellum 4-articulate; article 1 elongate and scale-like with setae on posterodorsal margin; articles 2–4 tapered and setose. Antennae 2 dorsal margin of peduncular article 1 and ventral margin of article 2 with short setae clusters; peduncular article 3 elongate with sparse small setae. Article 4 damaged and flagella lost in collection.

***Mouthparts***. (Fig. 12) ***Upper lip*** medial face asetose, with asymmetrical ventral margin. ***Mandible*** lacinia mobilis present on left and right side, highly asymmetric, left lacinia mobilis broad and multi-dentate, 22 robust setae on setal row. Right lacinia mobilis thin with broad projection at proximal end; 21 robust setae on setal row. Mandible incisors with strong posterodistal projection, slightly asymmetric; molar absent. Mandible palp 3-articulate, article 1 asetose, short; article 2 arched medially with 28 setae on medial margin; article 3 with many setae arranged in rows. ***Lower lip*** with distinct outer lobes present; mandibular process absent or severely reduced. ***Maxilla 1*** inner plate small with six apical plumose setae; outer plate arched medially with 14 robust apical setae with one large claw-like projection at the distal end. Maxilla 1 palp 2-articulate; article 1 subrectangular, setae on posterior; article 2 expanded distally with 16 apical and nine marginal robust setae, apical submargin with setae. ***Maxilla 2*** inner plate with 16 plumose setae along apical to medial margin, outer plate thin with three plumose setae along the apical margin. ***Maxilliped*** with inner and outer plates and palp; inner plate small and subtriangular with one apical plumose setae, one additional robust setae on the ventral margin; outer plate rounded, reaching base of palp article 2, with robust setae along medial to apical margin. Maxilliped palp long, 4-articulate; article 1 short, lateral margin with sparse short setae, medial margin with dense setae; article 2 longest article, similar pattern of setae as article 1; article 3 long, slightly shorter than article 2 with similar setae pattern; article 4 slender with strong claw-like projections on medial margin, tapered distally.

***Body***. Pereon with subrectangular pereonites, dorsal and ventral margin smooth; pleon with dorsal surface smooth; coxal gills present on gnathopod 2, pereopods 3–6; gills 2–4 elongate, gills 5 and 6 short and ovular; dorsal margins of urosomites 1 and 2 with single thorn-like projection each, pointed upward in urosomite 1, tapered distally in urosomite 2, urosomite 3 dorsal margin smooth.

***Gnathopods***. (Fig. 13A–F) ***Gnathopod 1*** coxa subrectangular and slightly bilobate, ventral margin straight; basis slightly arched distally, with sparse setae, length 1.78× width; ischium small and subquadrate, single clump of setae near base of merus on posterior margin; merus broad, concave along proximal margin, ventral margin with evenly separated clumps of setae; carpus broad and rounded, length 2.3× width, dorsal margin smooth with short sparse setae, lateral face asetose, posterior ventral submargin and medial face with dense clumps of setae; propodus narrower than carpus, posterior margin with dense setae, with one robust seta at ventral posterodistal corner; dactylus thin, ventral margin with setae, posterior margin with three strong projection near base. ***Gnathopod 2*** coxa subrectangular and slightly bilobate; basis straight and elongate, length 1.7× width, setae on posterior margin; ischium similar to gnathopod 1; merus more elongate than gnathopod 1 with numerous dense setae clumps on ventral submargin; carpus deep, subrectangular, length 2.3× width, dorsal margin smooth with sparse setae, lateral face asetose, ventral submargin and medial face densely setose; propodus with numerous, broadly plumose setae, otherwise similar to gnathopod 1; dactylus with majority of posterior margin asetose with five strong projections near base.

***Pereopods***. (Figs 13G–J, 14) ***Pereopod 3*** coxa ovular, slightly bilobate along dorsal margin, ventral submargin rounded with sparse setae; basis elongate, anterior and posterior margin setose, large clump of setae on posterodistal angle; ischium anterior margin concave, dense clump of setae on posterodistal margin; merus, carpus, propodus, and dactylus length ratio 1.00: 0.91: 0.37: 0.37; merus elongate, distal margin tapered, dorsal and ventral margins straight, posterior margin setose; carpus elongate, rounded; dorsal margin asetose, posterior margin with dense setae. Propodus elongate, thin, anterior margin asetose, posterior margin with short setae; dactylus slender with single robust seta near distal end. ***Pereopod 4*** similar to pereopod 3. ***Pereopod 5*** coxa subrectangular, tapered near proximal end, dorsal margin slightly convex, ventral margin straight, sparsely setose, length 2.4× width; basis broad and rectangular, length 3.4× width, posterior margin with many thin setae, anterior margin sparsely setose, posterodistal corner rounded; merus thin, anterior and posterior margin setose; medial face setose; carpus slightly shorter than merus, with similar setae arrangement. ***Pereopod 6*** coxa subrectangular, slightly tapered at distal end, ventral margin slightly concave, distal end sparsely setose; entire pereopod long and slender; basis broad, length 2.3× width, posterodistal corner rounded. ***Pereopod 7*** coxa slender and slightly tapered at distal end, ventral margin slightly concave, posterodistal margin with sparse setae, medial margin with large penile papillae faced medially.

***Pleon and urosome***. (Fig. 15) ***Epimeron*** setose on ventral submargin; epimeral plate 1 ventral margin curved with strong groove above the ventral margin, posterodistal corner pointed; epimeral plate 2 subrectangular, with strong groove above the ventral margin, posterodistal corner rounded; epimeral plate 3 subrectangular, posterodistal corner quadrate. ***Pleopods 1–3*** with paired coupling setae (retinacula) on medial distal margin of peduncle; coupling setae with hooks on inner margin; rami of similar length; inner ramus with numerous bifid (clothespin) setae on inner basal margin; ramus articles wide. ***Uropod 1*** peduncle longer than rami, 13 basofacial setae, distomedial projection strong; medial margin setose, sparse setae on lateral margin; outer ramus damaged, medial margin setose, lateral margin with few setae. ***Uropod 2*** peduncle triangular, about same length as rami, eight basofacial setae, distomedial peduncular projection similar to uropod 1; rami with setae along medial margins, sparse setae on lateral margin; outer ramus damaged. ***Uropod 3*** from holotype lost in collection. Female uropod 3 peduncle short and broad with sparse setae on the lateral margin and a row of plumose setae on the distomedial margin, rami ovular and flattened, lined with bases for long setae, most damaged or missing in collection, outer rami slightly tapered distally. ***Telson*** length 2.18× width, cleft extending 89% of length, lobes weakly tapered distally at last 1/3, apex of each lobe concave.

##### Sexual dimorphism.

Female specimens have a slender, setose 6 articulate accessory flagellum on antenna 1 (Fig. 11D). Female individuals also have a much stronger distomedial peduncular projection on uropod 1 than males (Fig. 15E), as well as shorter uropod rami. There is no significant difference in maximum sizes between male and female individuals.

##### Variable characters.

The number of basal projections on the dactylus of the gnathopods varied between specimens regardless of sex. The number of basal projections on gnathopod 1 varied from three to five, with four being the most common across examined specimens (see Fig. 13K for typical arrangement and placement). The number of basal projections on gnathopod 2 varied between four, five, and six, with five being the most common across examined specimens. The number of dactylus projections on gnathopod 2 is always greater or equal to that of gnathopod 1. The number of setae on the mandible setal row varies between specimens, with the left having 20–26 setae and right having 16–21 setae. The number of robust setae on the inner plate of maxilla 1 can vary between 7–10 setae.

##### Differential diagnosis.

*Princaxelia kahat* sp. nov. can be differentiated from most other *Princaxelia* species by the presence of six plumose setae on the inner plate of maxilla 1, with the sole exception of *P. magna* which also shows this feature. *Princaxelia kahat* sp. nov. is morphologically very similar to *P. magna*, with key differences seen in *Princaxelia kahat* sp. nov. being a longer antennae 1, telson lobes that are broader and tapered laterally, and an upper lip that is much blunter than that seen illustrated for *P. magna*.

##### Etymology.

The specific epithet *kahat* is derived from a Chamorro word meaning “to hunt stealthily, as if stalking or sneaking”, referring to the observed predatory behaviour of *Princaxelia*. This also reflects its habitat, as it hunts under the darkness of the hadal zone. The name is treated as an indeclinable noun in apposition.

##### Remarks.

The distinct morphological differences between *Princaxelia* species can be seen in Table 1, modified from Tomikawa et al. (2021). As seen in this table, the projection near the base of the dactylus is likely a diagnostic feature that should be noted for future *Princaxelia* captures. This was first suggested as a diagnostic feature of the genus in Tomikawa et al. (2021), and this description solidifies it as a distinct trait. This feature was not mentioned nor illustrated in the descriptions of *P. abyssalis* and *P. stephenseni*, and was also not noted in the review of the specimens by Lörz (2010). These are illustrated and mentioned briefly in the description of *P. magna*; however, the exact number is not specified and is obscured in the illustration of gnathopod 2 making this unclear. More specimens will need to be captured to confirm the presence or absence of this feature in those species.

**Table 1.** Morphology matrix of the genus *Princaxelia*.

**Species**	***Princaxelia abyssalis***	***Princaxelia jamiesoni***	***Princaxelia magna***	***Princaxelia marianaensis***	***Princaxelia stephenseni***	***Princaxelia malohi* sp. nov**.	***Princaxelia kahat* sp. nov**.
**Literature**	Dahl (1959)	Lörz (2010); Additional info from Tomikawa et al. (2021)	Kamenskaya (1977)	Tomikawa et al. (2021)	Dahl (1959); Additional info from Lörz (2010)	This study	This study
**Location**	Aleutian, Kuril – Kamchatka, Izu Bonin (Izu-Ogasawara), Yap, Japan, Philippine, Bougainville and Kermadec trenches	Kuril–Kamchatka, Japan and Izu-Ogasawara trenches	Yap Trench, dubious reports from Japan and Tonga trenches	Mariana Trench	North Atlantic: Southwest of Iceland	Tonga Trench	Mariana Trench
**Depth**	6,620–8,300 m	7,703–9,316 m	7,190–7,250 m	5,683 m	1,505 m	8,200–8,350 m	8,000–8,964 m
**Maximum known size**	Male: 21 mm; Female: 32 mm	Male: 57 mm; Female: 61 mm	Male: 52 mm; Female: N/A	Male: N/A; Female: 23.9 mm	Male: 10 mm; Female: 11 mm	Male: 39.5 mm; Female: N/A	Male: 59.3 mm; Female: 61.8 mm
**Epimeral plate 3 posterodistal corner**	Rounded	Quadrate	Quadrate	Quadrate	Weakly Rounded	Quadrate	Quadrate
**Dorsal projections on urosomites 1 and 2**	Unknown	Pointing toward distal end	Pointing upright	Pointing toward distal end	Pointing toward distal end	Pointing toward distal end	Urosomite 1 pointed upright; Urosomite 2 pointed distally
**Upper lip**	Unknown	Slightly asymmetrical	Strongly symmetrical	Strongly asymmetrical	Slightly asymmetrical	Strongly asymmetrical	Slightly asymmetrical
**Maxilla 1 palp article 2**	Expanded; less than 14 apical robust setae	Expanded; 25 apical robust setae	Expanded; ~10 apical robust setae	Expanded; 9 apical robust setae	Not expanded; 7 apical robust setae	Expanded; 13 apical robust setae	Expanded; 14 apical robust setae
**Maxilla 1 inner plate**	1 plumose seta	1 plumose seta	6 plumose setae	1 plumose seta	1 plumose seta	2 plumose setae	6 plumose setae
**Maxilla 2 outer plate**	3 apical plumose setae	3 apical plumose setae	3 apical plumose setae	3 apical plumose setae	3 apical plumose setae	6 apical plumose setae	3 apical plumose setae
**Gnathopods 1 and 2 dactyli basal projections**	Unknown	7–9 projections	4 projections	3 projections	Unknown	1 projection	3–5 (4 most common) projections on gnathopod 1; 4–6 (5 most common) projections on gnathopod 2
**Coxa 5 dorsal margin**	Highest at proximal end	Straight	Convex	Highest at proximal end	Straight/convex	Straight	Highest at proximal end
**Coxa 5 distal margin**	Rounded	Rounded	Slightly pointed	Rounded	Straight	Slightly pointed	Rounded
**Coxa 7 ventral margin**	Straight	Slightly concave	Slightly concave	Shallowly concave	Straight	Shallowly concave	Shallowly concave
**Uropod distomedial corner projection**	Unknown	Very strong	Unknown	Strong	Unknown	Weak	Very strong in females, average in males
**Telson lobe**	Thin; Uniformly tapering distally	Broad; Tapering from distal 1/3	Thin; Tapering from distal 1/3	Thin; Uniformly tapering distally	Unknown	Broad; Weakly tapering distally	Broad; Tapering from distal 1/3

An additional feature which may be diagnostic is the distomedial corner of the uropods, which is very weak in *Princaxelia malohi* sp. nov. *Princaxelia kahat* sp. nov. shows a much stronger projection in female individuals than males, but the projection in male individuals is stronger than that seen in male individuals of *Princaxelia malohi* sp. nov. This feature appears very strong in the other described species with illustrations, specifically in those illustrated from female individuals. This is likely a sexually dimorphic feature and should be noted for future *Princaxelia* descriptions.

### Key to the genus *Princaxelia*

Updated dichotomous key to the genus *Princaxelia*, adapted from Tomikawa et al. (2021).

**Table:** 

1	Palp article 2 of maxilla 1 expanded	**2**
–	Palp article 2 of maxilla 1 not expanded	***P. stephenseni* Dahl, 1959**
2	Maxilla 1 inner plate with two or less plumose setae	**3**
–	Maxilla 1 inner plate with more than two plumose setae	**6**
3	Coxa 7 ventral margin concave	**4**
–	Coxa 7 ventral margin straight	***P. abyssalis* Dahl, 1959**
4	Maxilla 1 palp article 2 with less than 20 apical robust setae	**5**
–	Maxilla 1 palp article 2 with more than 20 apical robust setae	***P. jamiesoni* Lörz, 2010**
5	Maxilla 1 inner plate with single plumose setae, gnathopod dactyli with 3 basal projections	***P. marianaensis* Tomikawa & Watanabe in Tomikawa et al. 2021**
–	Maxilla 1 inner plate with two plumose setae, gnathopod dactyli with single basal projection	***Princaxelia malohi* sp. nov**.
6	Antennae 1 shorter than half the length of body, strongly asymmetrical, sharp upper lip, thin telson lobes	***P. magna* Dahl, 1959**
–	Antennae 1 longer than half the length of body, weakly asymmetrical, blunt upper lip, broad telson lobes	***Princaxelia kahat* sp. nov**.

### *In situ* behaviours

**Figure 16.**
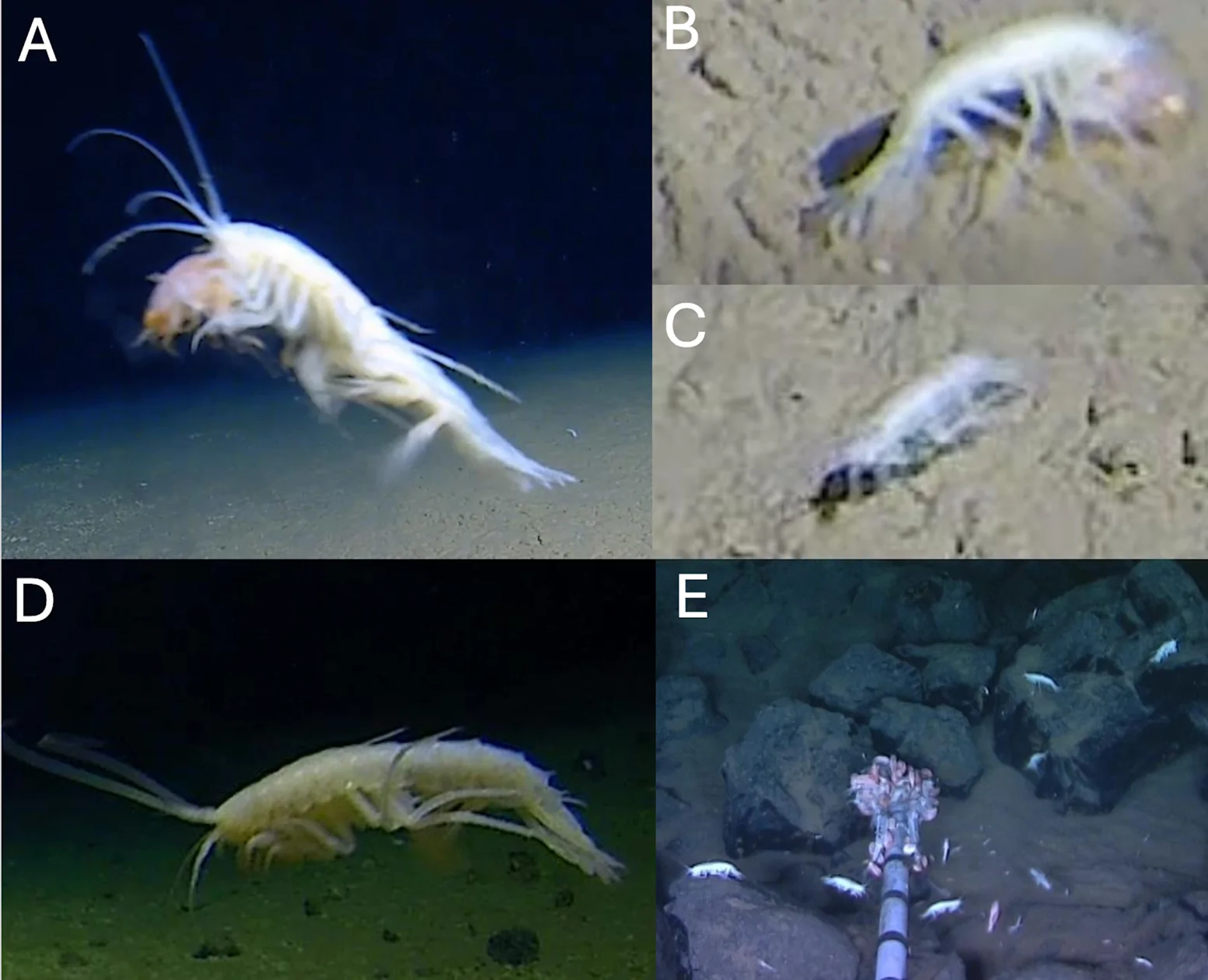
**A–C**. *In situ* observations of *Princaxelia* from 8,200 m in the Tonga Trench; **A**. *Princaxelia* individual swimming with prey item (amphipod; *Hirondellea dubia*); **B**. Individual positioning prey for feeding; **C**. *Princaxelia* individual in motion across seafloor, showing trailing pereopods six and seven; **D**. Free swimming posture captured from an individual in the Mariana Trench; **E**. Aggregation of seven individuals at 8,098 m around bait on a boulder field in the Mariana Trench.

*Princaxelia* individuals were observed at 8,200 meters in the Tonga Trench capturing amphipod prey using the anterior appendages, holding prey between the gnathopods and maxillipeds while swimming, and then restraining prey against the sediment surface during consumption (Fig. 16A–C; https://doi.org/10.5281/zenodo.20756281). The typical free-swimming posture of the genus was also observed, showing a raised pereopod 5 (Fig. 16D). Large aggregations of *Princaxelia* were also observed on lander video footage in both the Tonga and Mariana (Fig. 16E) Trenches, providing better views on their behaviours.

### Pairwise distances

Pairwise distances are presented in Suppl. material 2. In *Princaxelia malohi* sp. nov. no intraspecific variation was detected among the newly generated 16S rRNA sequences, with maximum uncorrected *p*-distances and Kimura 2-parameter (K2P) distance both equal to 0.00%. In contrast, *Princaxelia kahat* sp. nov. *exhibited* low intraspecific divergence, with maximum uncorrected *p*-distance and maximum K2P distance of 0.25%. Pairwise divergence between *Princaxelia malohi* sp. nov. and *Princaxelia kahat* sp. nov. was substantially greater, ranging from 15.76–16.42% for uncorrected *p*-distance and 17.71–18.54% K2P distance. For COI, a single newly generated *Princaxelia malohi* sp. nov. sequence was obtained, precluding estimation of within-species genetic distances. Pairwise comparisons with publicly available *Princaxelia* sequences showed uncorrected *p*-distance of 24.05% and K2P distance of 29.61% for *Princaxelia* sp. (PX720326), and 15.07% uncorrected p-distance from *P. marianaensis* (LC719249) of 15.97% and 16.90% for K2P.

## Discussion

This study provides descriptions of two new predatory amphipods of the genus *Princaxelia* from hadal depths. *Princaxelia malohi* sp. nov. collected from 8,200–8,350 m water depth in the Tonga Trench, and *Princaxelia kahat* sp. nov. collected from between 8,000–8,964 m in the Mariana Trench. These descriptions increase the number of formally described species in the genus to seven and provide both morphological diagnoses and mitochondrial DNA barcodes for 16S and COI, where amplification was successful. The addition of genetic data is particularly important for *Princaxelia*, for which sequence data remains limited. At present, only *P. marianaensis* has been described with an associated COI sequence, although additional unidentified *Princaxelia* sequences are available from broader amphipod genetic studies (Jażdżewska and Mamos 2019; Jażdżewska et al. 2026). The new sequences generated here therefore provide an important reference point for future work on the taxonomy, diversity, and biogeography of *Princaxelia* and other deep-sea pardaliscid amphipods.

### Morphological taxonomy

The taxonomy of *Princaxelia* remains difficult to resolve because several described species are based on incomplete or poorly preserved material. No complete type specimens are available for *P. stephenseni*, *P. abyssalis*, or *P. magna*. For *P. stephenseni*, only damaged mouthparts remain, while *P. abyssalis* is represented only by dissected appendages. The repository of the type specimen of *P. magna* was not stated in the original description, and previous attempts to locate it were unsuccessful (Lörz 2010). As a result, the descriptions of these species remain incomplete, despite the additional notes and revisions provided by Lörz (2010). This uncertainty is particularly relevant for *Princaxelia kahat* sp. nov., which closely resembles the published description and illustrations of *P. magna* in several morphological features. However, some illustrations of *P. magna* are unclear and key diagnostic information is missing, preventing confident assignment of the Mariana Trench material to that species. The uncertain status of the *P. magna* holotype, together with dubious reports of this species from the Tonga and Japan trenches (Beliaev and Brueggeman 1989), means that *Princaxelia kahat* sp. nov. is here treated as a distinct species rather than as a range extension of *P. magna*.

Scanning electron microscopy images also remain rare for deep-sea amphipods and is especially limited for Pardaliscidae. Martin et al. (1993) reported scale-like structures across the body surface of a pardaliscid amphipod and suggested that similar structures may occur more widely within the family. In contrast, SEM examination of *Princaxelia malohi* sp. nov. showed a lack of prominent surface scales and instead have a smooth exoskeletal surface (Fig. 6). A faint surface pattern is visible only along the ventral margin of the gnathopod dactylus in *Princaxelia malohi* sp. nov. (Fig. 6D). Although the taxonomic value of this character is currently uncertain, the presence or absence of surface sculpturing may become useful as SEM is more widely applied to deep-sea amphipod taxonomy.

### Biogeography

Current distribution records suggest that *Princaxelia* may be more widespread and more diverse than formal taxonomy presently indicate. Although *P. abyssalis* has been reported from eight trench systems, it has only been formally described from the Kermadec Trench. Reports from other trench systems generally lack sufficient morphological detail or physical specimens to confirm these identifications and may instead represent undescribed species. This interpretation is supported by the absence of recent confirmed records matching the description of *P. abyssalis* from those regions, and was speculated in (Jamieson et al. 2022). Additional evidence for a wider distribution of the genus comes from unidentified specimens resembling *Princaxelia* observed in video footage from the Java Trench at 5,760–6,957 m (Jamieson et al. 2022), and from a damaged specimen collected from the Diamantina Fracture Zone at 7,009 m (Jamieson and Weston 2023). Continued sampling, particularly when paired with high-resolution imaging and DNA sequencing, will be necessary to determine whether these records represent known species, range extensions, or additional undescribed taxa.

The discovery of *Princaxelia kahat* sp. nov. provides the first evidence of more than one *Princaxelia* species occurring within a single trench system. Both *P. marianaensis* and *Princaxelia kahat* sp. nov. are known from the Mariana Trench, but they occur in markedly different settings. *Princaxelia marianaensis* was collected from cold seep habitats at the Shinkai Seep Field at 5,683 m (Tomikawa et al. 2021), whereas *Princaxelia kahat* sp. nov. was collected from boulder-field habitats below 8,000 m. This suggests possible niche partitioning by depth, habitat type, or both. However, the true distributions and ecological ranges of these species remain poorly constrained, and substantially more material will be required to test whether these patterns reflect habitat specialization, depth stratification, or under sampling.

The Atlantic record of *P. stephenseni* remains especially intriguing. This species is currently the only representative of *Princaxelia* identified from the Atlantic Ocean and occurs at a comparatively shallow depth relative to most other members of the genus (Stephensen 1931). This may indicate that additional *Princaxelia* species remain undiscovered in Atlantic deep-sea ecosystems, or that the genus is influenced by temperature as well as depth, with shallower occurrences possible in colder polar or subpolar environments. At present, however, the limited material available for *P. stephenseni* prevents strong conclusions about its relationship to other species in the genus.

### Behaviour and ecology

*In situ* video footage from the Tonga Trench provides new insight into the behaviour and ecology of *Princaxelia*. Similar predatory behaviour to what has been captured in these observations was previously observed in baited video footage from the Tonga Trench, where the individuals were identified as *P. abyssalis* (Jamieson et al. 2012). Given the new material described here, those observations may instead represent *Princaxelia malohi* sp. nov. The observed behaviour is consistent with an active benthopelagic predator that exploits dense aggregations of scavenging amphipods at baited deployments.

The locomotory behaviour of *Princaxelia* also contrasts with previous suggestions that pardaliscid amphipods are primarily pelagic (Bousfield 1979). In the available footage, individuals were repeatedly observed sitting on the sediment with pereopods 5–7 outstretched, gliding over the sediment using pereopods 3 and 4 with assistance from the pleopods, and swimming above the seafloor. Pereopods 6 and 7 did not appear to be used directly for locomotion. During free swimming, *Princaxelia* adopted a distinctive posture in which pereopods 3 and 4 were tucked beneath the pereon, pereopod 5 was angled upward from the ischium, and pereopods 6 and 7 trailed behind the body (Fig. 16 C, D). This combination of sediment-associated ambush behaviour and short-distance swimming is similar to the predatory strategies described for morphologically comparable amphipods in the family Eusiridae (Klages and Gutt 1990).

Across all available footage, *Princaxelia* individuals were observed feeding on other amphipods. At baited deployments, they appeared to exploit the high local abundance of scavenging amphipods, particularly *Hirondellea* sp. in the Mariana Trench (Fig. 16E). However, previous gut-content analysis indicates that members of the genus may also consume, or incidentally ingest, sponge and echinoid material (Kamenskaya 1981). This suggests that while amphipods may be the primary observed prey in baited-camera settings, the broader diet of *Princaxelia* may be more diverse than video observations alone indicate.

The relatively high abundance of *Princaxelia* in some hadal observations suggests that these predators may play an important ecological role in trench food webs (Dasgupta et al. 2024). In the Tonga Trench at 8,200 m, up to five individuals were observed simultaneously, while in the Mariana Trench at 8,098 m, up to seven individuals were observed at the same baited deployment. High local abundance is also supported by epibenthic sledge material from the Kuril–Kamchatka Trench, where *Princaxelia* individuals made up approximately 60% of the amphipods collected at 9,427 m (Brandt et al. 2016). These observations indicate that *Princaxelia* may be locally abundant where prey densities are high, particularly around baited food falls or natural aggregations of scavenging amphipods.

Despite their role as conspicuous predators of amphipods, *Princaxelia* may also be important prey for larger hadal predators. Individuals have been observed being consumed by snailfish through suction feeding at upper hadal depths (Dasgupta et al. 2024). This predator-prey relationship is notable because fishes appear to reach their lower depth limit at approximately 8,300 m, close to the depth range occupied by several *Princaxelia* species, including the two new species described here. Below this depth, where fishes are absent, *Princaxelia* may occupy a particularly important predatory role within hadal amphipod communities.

## Conclusions

The formal descriptions of *Princaxelia malohi* sp. nov. and *Princaxelia kahat* sp. nov. are presented expanding the known diversity of large predatory amphipods inhabiting the hadal zone and highlighting how much remains unresolved about predator-prey dynamics below 8,000 m. Together, the new morphological descriptions, genetic data, and *in situ* behavioural observations show that *Princaxelia* is not simply a rare taxonomic curiosity, but a potentially important predator within hadal amphipod communities. Continued collection of specimens, combined with high-resolution imaging, molecular data, and video observations, will be essential for resolving the true diversity, distribution, and ecological role of this genus. These findings reinforce that even among the most conspicuous animals of the deepest trenches, fundamental aspects of taxonomy, behavior, and food-web structure remain poorly understood.

## Supplementary Material

XML Treatment for Princaxelia malohi

XML Treatment for Princaxelia kahat

## Additional information

### Artificial Intelligence (AI) use

The authors accept full responsibility for the content of the manuscript, including the disclosure of any use of AI.

No AI tools were used in the preparation of this manuscript.

### Author ORCIDs

G.A. Duffy https://orcid.org/0009-0002-6972-6013

J.A. Wainwright https://orcid.org/0000-0001-8598-5248

B.C. Gonzalez https://orcid.org/0000-0001-6968-2677

T. Bond https://orcid.org/0000-0001-6064-7015

A.J. Jamieson https://orcid.org/0000-0001-9835-2909

### Data availability

Data available upon reasonable request. Supplementary videos available online at: https://doi.org/10.5281/zenodo.20756281.

## Supplementary materials

Supplementary material 1 GenBank accession numbers Data type xlsx Explanation note **table S1**. GenBank accession numbers for *Princaxelia kahat* sp. nov. and *Princaxelia malohi* sp. nov. and those included in the COI and 16S alignments. https://binary.pensoft.net/file/1745927This dataset is made available under the Open Database License (http://opendatacommons.org/licenses/odbl/1.0/). The Open Database License (ODbL) is a license agreement intended to allow users to freely share, modify, and use this Dataset while maintaining this same freedom for others, provided that the original source and author(s) are credited. Grady A. Duffy, Jennifer A. Wainwright, Brett C. Gonzalez, Todd Bond, Alan J. Jamieson

Supplementary material 2 Pairwise distances Data type xlsx Explanation note **table S2**. Uncorrected p-distances and Kimura two-parameter (K2P) distances for COI and 16S rRNA. https://binary.pensoft.net/file/1745928This dataset is made available under the Open Database License (http://opendatacommons.org/licenses/odbl/1.0/). The Open Database License (ODbL) is a license agreement intended to allow users to freely share, modify, and use this Dataset while maintaining this same freedom for others, provided that the original source and author(s) are credited. Grady A. Duffy, Jennifer A. Wainwright, Brett C. Gonzalez, Todd Bond, Alan J. Jamieson
